# Induction of a torpor-like hypothermic and hypometabolic state in rodents by ultrasound

**DOI:** 10.1038/s42255-023-00804-z

**Published:** 2023-05-25

**Authors:** Yaoheng Yang, Jinyun Yuan, Rachael L. Field, Dezhuang Ye, Zhongtao Hu, Kevin Xu, Lu Xu, Yan Gong, Yimei Yue, Alexxai V. Kravitz, Michael R. Bruchas, Jianmin Cui, Jonathan R. Brestoff, Hong Chen

**Affiliations:** 1 Department of Biomedical Engineering, Washington University in St. Louis, Saint Louis, MO USA; 2 Department of Pathology and Immunology, Washington University School of Medicine, Saint Louis, MO USA; 3 Department of Psychiatry, Washington University School of Medicine, Saint Louis, MO USA; 4 Departments of Anesthesiology and Pain Medicine, Pharmacology, and Bioengineering, Center for Neurobiology of Addiction, Pain, and Emotion, University of Washington, Seattle, WA USA; 5 Department of Radiation Oncology, Washington University School of Medicine, Saint Louis, MO USA; 6 Department of Neurosurgery, Washington University School of Medicine, St. Louis, MO USA; 7 Division of Neurotechnology, Washington University School of Medicine, Saint Louis, MO USA

**Keywords:** Biological techniques, Neuroscience, Metabolism

## Abstract

Torpor is an energy-conserving state in which animals dramatically decrease their metabolic rate and body temperature to survive harsh environmental conditions. Here, we report the noninvasive, precise and safe induction of a torpor-like hypothermic and hypometabolic state in rodents by remote transcranial ultrasound stimulation at the hypothalamus preoptic area (POA). We achieve a long-lasting (>24 h) torpor-like state in mice via closed-loop feedback control of ultrasound stimulation with automated detection of body temperature. Ultrasound-induced hypothermia and hypometabolism (UIH) is triggered by activation of POA neurons, involves the dorsomedial hypothalamus as a downstream brain region and subsequent inhibition of thermogenic brown adipose tissue. Single-nucleus RNA-sequencing of POA neurons reveals TRPM2 as an ultrasound-sensitive ion channel, the knockdown of which suppresses UIH. We also demonstrate that UIH is feasible in a non-torpid animal, the rat. Our findings establish UIH as a promising technology for the noninvasive and safe induction of a torpor-like state.

## Abstract (web summary)

Using ultrasound to activate noninvasively specific neurons in the hypothalamus, a temporary hypothermic and hypometabolic state is induced in rodents.

## Main

Torpor, like hibernation, is a physiological state in which mammals actively suppress metabolism, reduce body temperature and slow down other live processes to conserve energy and survive fatal conditions and cold environmental temperatures^1^. The concept of inducing torpor-like hypothermia and hypometabolism by artificial means was initially proposed in 1960 as a biomedical solution to reduce energy consumption during long-term human spaceflight^2,3^. Torpor-like hypothermia and hypometabolism could also increase the survival probability of patients under life-threatening conditions (for example, heart attack or stroke) by slowing metabolism and disease progression^4^.

Noninvasive and safe induction of a torpor-like state has been considered science fiction confined to movies and novels^5,6^. Despite several decades of research, it still has not been attained. The original concept proposed that hibernation is regulated by endogenous blood substances^7^ and extensive efforts were devoted to searching for endogenous substances that induce a torpor-like state through systemically suppressing metabolism^8,9^. It is now believed that torpor is controlled by the central nervous system (CNS) to precisely coordinate numerous functions^10–12^. It was reported that direct intracranial injection of pharmaceutical agents targeting the CNS pathways induced a deep hypothermic state resembling natural torpor^13,14^. Recent groundbreaking studies identified several neuronal populations in the hypothalamic POA that regulate torpor and hibernation in rodents^11,12,15^. Genetic engineering of these neuronal populations for optogenetic and chemogenetic manipulation obtained critical behavioral and physiological features of torpor/hibernation in mice^11,12,15^. Although these technological advances in inducing a torpor-like state are promising, these approaches require surgical intervention or genetic engineering, limiting the broad application of these approaches and translation to humans.

**Fig. 1:**
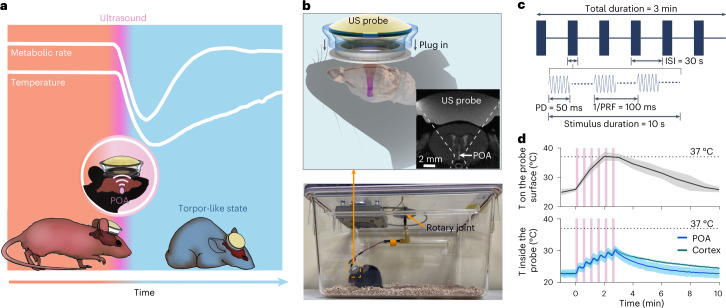
Ultrasound device for inducing a torpor-like hypothermic and hypometabolic state. **a**, Illustration of ultrasound (US)-induced torpor-like state. **b**, Illustration of the wearable US probe (top). The probe was plugged into to a baseplate that was glued on the mouse’s head. MRI of the mouse head with the wearable US probe shows that ultrasound was noninvasively targeted at the POA (insert). Photograph of a freely moving mouse with the wearable US probe attached is shown at the bottom. **c**, Illustration of the US stimulation waveform used in this study. ISI, inter-stimulus interval; PD, pulse duration; PRF, pulse repetition frequency. **d**, Calibration of the temperature (T) rise on the surface (top) and inside (bottom) the US probe. The temperature inside the probe was measured between the piezoelectric material and the mouse head when US probes were targeted at the POA or the cortex. Cortex was selected as an off-target control. US sonication is indicated by the pink bars. *n* = 6 mice (top) and *n* = 4 mice (bottom). Solid lines and shadows denote the mean ± s.e.m. Source data

Ultrasound is the only available energy form that can noninvasively penetrate the skull and focus on any location within the brain with millimeter precision and without ionizing radiation^16,17^. These capabilities, along with its safety, portability and low cost, have made ultrasound a promising technology for neuromodulation in small animals^18,19^, non-human primates^20,21^ and humans^16,22^, although its mechanism remains elusive. Here, we report noninvasive, precise and safe induction of a torpor-like state in mice through remote ultrasound stimulation at the POA (Fig. 1a). We discovered that this UIH was associated with ultrasonic activation of the TRPM2 ion channel expressed in torpor-associated POA neurons. We uncovered the potential involvement of the dorsomedial hypothalamus as a downstream brain region and brown adipose tissue as an effector tissue in the regulation of UIH. We also demonstrated that ultrasound stimulation at POA successfully induced hypothermia in rats, a non-torpid animal.

## Results

### Ultrasound at POA triggers hypothermia and hypometabolism

**Fig. 2:**
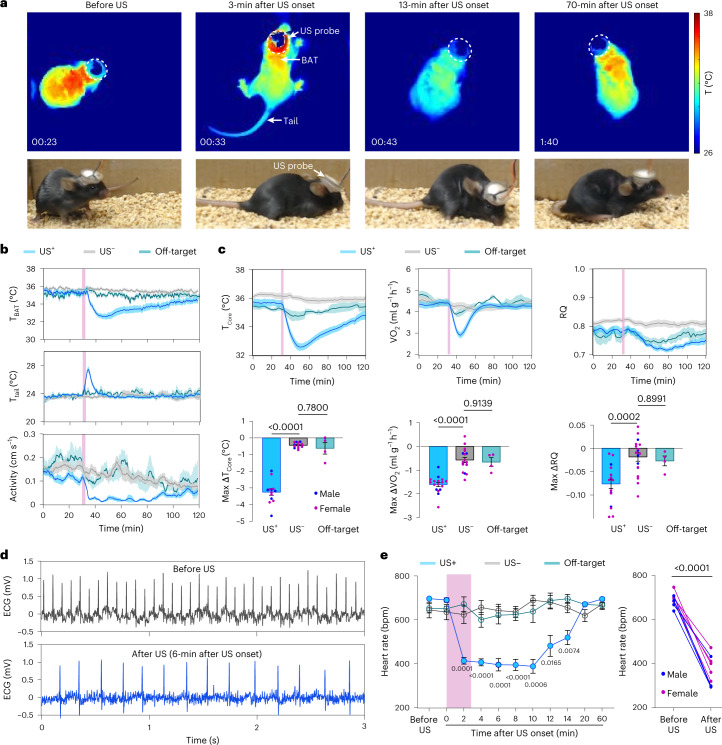
Ultrasound stimulation of POA induces hypothermia and hypometabolism. **a**, Infrared thermal images (top) and photos (bottom) of a mouse receiving US stimulation at POA. The US transducer probe is marked by dotted circles. **b**, BAT temperature (T_BAT_), tail temperature (T_tail_) and physical activity of mice with US stimulation at POA (US^+^, *n* = 12 mice) compared to two control groups: mice without US sonication (US^–^, *n* = 12 mice) and mice with US stimulation at the cortex as an off-target control (off-target, *n* = 6 mice). US sonication is indicated by the pink bars. **c**, Core body temperature (T_core_), metabolic rate (VO_2_) and RQ (VCO_2_/VO_2_) for the US^+^ (*n* = 13 mice for T_core_ and *n* = 17 mice for VO_2_ and RQ), US^–^ (*n* = 14 mice for T_core_ and *n* = 18 mice for VO_2_ and RQ) and off-target groups (*n* = 4 mice for T_core_, VO_2_ and RQ) (top, from left to right). Max ΔT_core_ (lowest T_core_ – mean T_core_ before US), max ΔVO_2_ (lowest VO_2_ – mean VO_2_ before US) and max ΔRQ (lowest RQ – mean RQ before US) (bottom, from left to right). Male and female mice are represented as blue and pink dots, respectively. **d**, Representative ECG traces in mouse before (top) and after US stimulation (bottom). **e**, Heart rate before and after US stimulation for the US^+^ (*n* = 9 mice), US^–^ (*n* = 5 mice) and off-target (*n* = 5 mice, targeting at the hippocampus) groups (left). No significant difference was found between the off-target and the US^–^ group throughout the entire recording time. Comparison of heart rate before US stimulation and the lowest heart rate achieved by US stimulation at POA within 10 min after US stimulation (right). bpm, beats per minute. Solid lines and shadows denote the mean ± s.e.m. Error bars denote s.e.m. Each dot represents one mouse. *P* values were calculated using a one-way analysis of variance followed by Dunnett’s post hoc test in **c** and **e** (left) comparing the US^+^ and off-target groups to the US^–^ group, respectively and a two-tailed paired *t*-test in **e** (right). Source data

**Extended Data Fig. 1:**
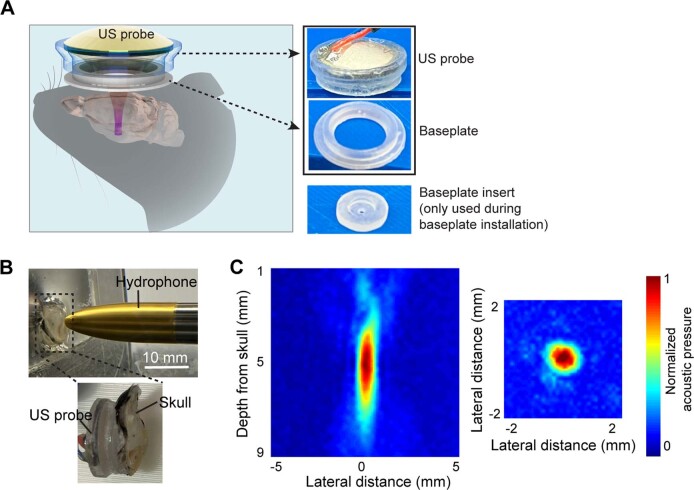
Experimental setup. (**A**) Illustration of the wearable ultrasound transducer. The transducer was attached to a baseplate that was glued on the mouse’s head. Photos of the ultrasound probe (US probe) and the baseplate. A photo of the baseplate insert is also provided. The baseplate insert with a hole in the center was used during baseplate installation to guide the positioning of the baseplate. The insert was removed after the baseplate installation. (**B**) The experimental setup for measuring the acoustic pressure fields generated by the ultrasound probe using a hydrophone in a degassed water tank. Ex vivo mouse skull with the baseplate was placed in front of the ultrasound probe. (**C**) The measured acoustic pressure fields in the axial focal plane and lateral focal plane in the presence of the mouse skull. The focal region size measured by the full-width-at-half-maximum was 3.8 mm in the axial direction and 0.8 mm in the lateral direction.

Natural torpor is characterized by key features of hypothermia, hypometabolism and hypoactivity^11,23^. We designed a ‘plug-and-play’ wearable ultrasound transducer (Fig. 1b and Extended Data Fig. 1) to remotely deliver ultrasound to the POA region of freely moving mice that had ad libitum access to food and water. We first recorded mouse body temperature change by a thermal infrared camera and found a profound decrease in skin temperature in the interscapular area where brown adipose tissue (BAT) resides (T_BAT_) (Fig. 2a and Supplementary Video 1), during and after ultrasound stimulation (1.6 MPa in acoustic pressure 3.2 MHz in frequency, 50 ms in pulse duration, 10 Hz in pulse repetition frequency, 10 s in stimulus duration, 30 s in inter-stimulus interval and 6 in total number of stimuli; Fig. 1c). This observation of a temperature decrease in BAT was synchronized with an increase in tail temperature (Fig. 2a,b). Two primary mechanisms underlying thermoregulation in rodents are BAT thermogenesis (which enables non-shivering heat production) and tail vasodilation (which enables heat dissipation). These findings indicated that ultrasound stimulation of POA in mice suppressed heat production and initiated heat loss to induce hypothermia. Video recordings of animal behavior showed that ultrasound stimulation of the POA also was correlated with a reduction in locomotor activity (Fig. 2b).

**Extended Data Fig. 2:**
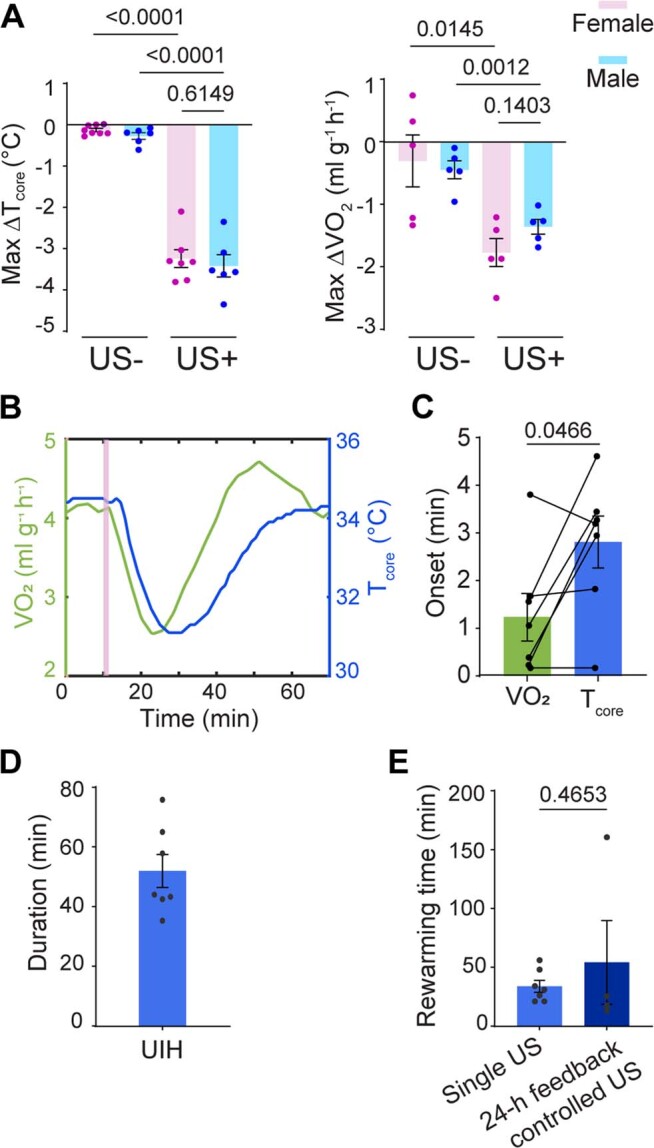
Characterization of the UIH. (**A**) UIH-induced core body temperature change (max ΔT_core_) and metabolic rate change (max ΔVO_2_) in female (pink) and male (blue) mice. In the left plot, n = 6 male mice in both US^+^ and US^−^ groups and n = 7 female mice in US^+^ and 8 female mice in US^−^ group. In the right plot, n = 5 mice in all groups. (**B**) Temporal correlation of VO_2_ and T_core_ induced by ultrasound stimulation. US sonication is indicated by the pink bar. (**C**) Comparison of VO_2_ and T_core_ onset time. N = 7 mice. (**D**) Quantification of the duration of the hypothermic state. N = 7 mice. (**E**) Quantification of the rewarming time (from the lowest T_core_ to 34 °C) in groups receiving single US stimulation and receiving long-duration feedback-controlled ultrasound stimulation. N = 7 mice for the single US stimulation group and 4 mice for the feedback-controlled ultrasound stimulation group. Error bars denote s.e.m. Each dot represents one mouse. P values were calculated using an unpaired two-tailed t-test in (A and E) and a paired two-tailed t-test in C. Source data

**Extended Data Fig. 3:**
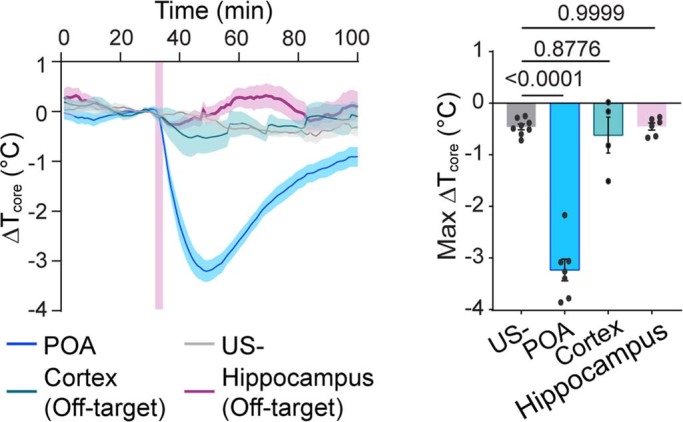
Change of the core body temperature and metabolic rate in the off-target groups. Change of the core body temperature relative to the baseline (ΔT_core_) and the calculated max ΔT_core_ for the four groups, including the group without ultrasound stimulation (US^−^), the group receiving ultrasound stimulation at the POA (POA), the off-target control group receiving ultrasound at the cortex (Cortex), and the off-target control group receiving ultrasound at the ventral hippocampus (Hippocampus). In the right figure, max ΔT_core_ was calculated as lowest T_core_ – mean T_core_ before US. US sonication is indicated by the pink bar. Solid lines and shadows denote the mean ± s.e.m. Error bars denote s.e.m. Each dot represents one mouse. N = 8 mice in the US^−^group, 7 mice in the POA group, 4 mice in the Cortex group and 6 mice in the Hippocampus group. P values were calculated using a one-way ANOVA followed by Dunnett’s post-hoc test comparing each group to US^−^. Source data

**Extended Data Fig. 4:**
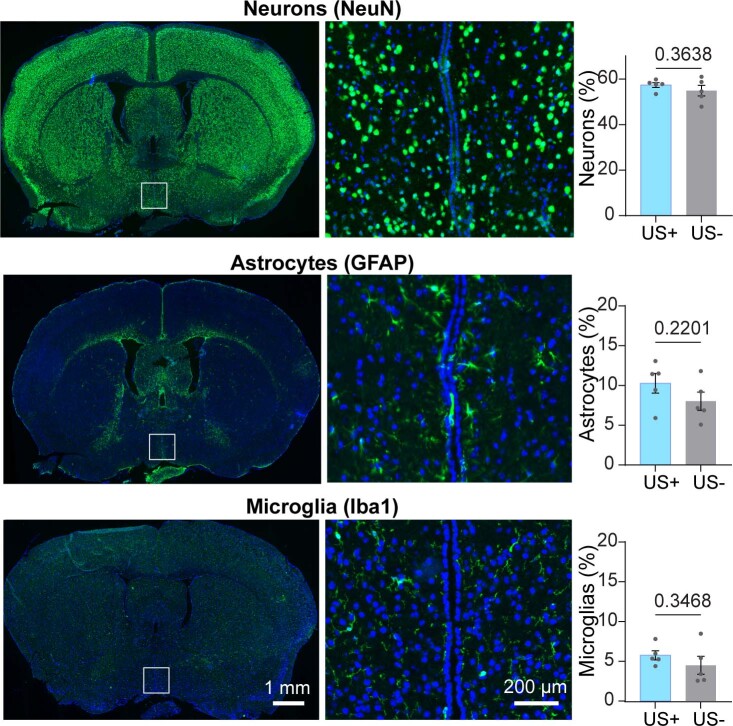
Safety assessment of ultrasound stimulation. Evaluation of the morphology and numbers of neurons, astrocytes, and microglia after ultrasound stimulation at the POA. Error bars denote s.e.m. Each dot represents one mouse. N = 5 mice. P values were calculated using the unpaired two-tailed t-test. Source data

**Extended Data Fig. 5:**
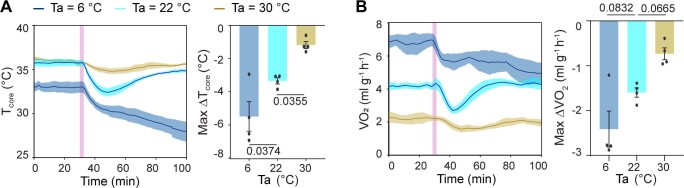
UIH in different ambient temperatures. (**A**) Core body temperature (T_core_) and (**B**) metabolic rate (VO_2_) for mice receiving ultrasound stimulation in different ambient temperatures (6 °C, 22 °C, and 30 °C). US sonication is indicated by the pink bars. Solid lines and shadows denote the mean ± s.e.m. Error bars denote s.e.m. Each dot represents one mouse. Max ΔT_core_ and max VO_2_ were calculated by computing the difference between the minimum value obtained within the 70 min period following the start of ultrasound and the baseline value which was defined as the mean body temperature of the 5-min window occurring 1 minute prior to the ultrasound stimulation. N = 4 mice for all the groups. P values were calculated using a one-way ANOVA followed by Dunnett’s post-hoc test comparing to the 22 °C ambient temperature group. Source data

We then placed mice in metabolic cages, which allowed us to assess their metabolism before, during and after ultrasound stimulation of the POA by analyzing the oxygen consumption rates (VO_2_) and respiratory quotient (RQ = VCO_2_ / VO_2_)^12^. These mice were implanted with wireless temperature sensors in their abdominal cavity to simultaneously measure the core body temperature (T_core_). Ultrasound stimulation led to a marked decrease in core body temperature (max ∆T_core_ = −3.26 ± 0.19 °C) (Fig. 2c) and VO_2_ (by 36.61 ± 1.74%) (Fig. 2c). These effect sizes were similar to the previously reported fasting-induced torpor in mice^11,24^. Ultrasound suppressed oxygen consumption before the fall in body temperature (Extended Data Fig. 2b,c), suggesting active inhibition of metabolism rather than a secondary effect of hypothermia. The duration of T_core_ reduction below body temperature was 51.98 ± 5.52 min by UIH (Extended Data Fig. 2d), which was comparable to natural torpor^24,25^. Ultrasound also decreased the RQ to 0.72 ± 0.01 (Fig. 2c). An RQ close to 0.7 indicates that UIH switched energy substrate utilization from a mixture of carbohydrates and fat to reliance on fat oxidation, which has been established as a common feature of torpid animals^26^. Mice undergoing UIH spontaneously arose from the hypothermic and hypometabolic state to a normal condition, which is also a critical feature of natural torpor^5,11,12^. Electrocardiogram (ECG) recordings showed that UIH decreased the heart rate by 47.3%, which was significantly lower than that before ultrasound stimulation and the group without ultrasound stimulation (Fig. 2d,e). These physiological states were not observed in mice without ultrasound stimulation or with ultrasound stimulation at off-target locations (cortex and ventral hippocampus) (Fig. 2b–e and Extended Data Fig. 3), verifying that the ultrasound-induced torpor-like state was evoked by POA-specific stimulation, instead of non-specific effects, such as heat produced by ultrasound piezoelectric material (Fig. 1d). Immunohistology examination of mouse brains after ultrasound treatment did not find any visible damage or inflammation within the brain (Extended Data Fig. 4). All the above experiments were performed at room temperature (~22 °C). We repeated the experiment in cold (6 °C) and thermoneutral (30 °C) ambient temperature and found that hypothermia and hypometabolism were successfully evoked (Extended Data Fig. 5). The effect size of UIH increased as the ambient temperature decreased (Extended Data Fig. 5), suggesting that the effect of UIH could be modulated by the ambient temperature.

### UIH is precisely controllable

**Fig. 3:**
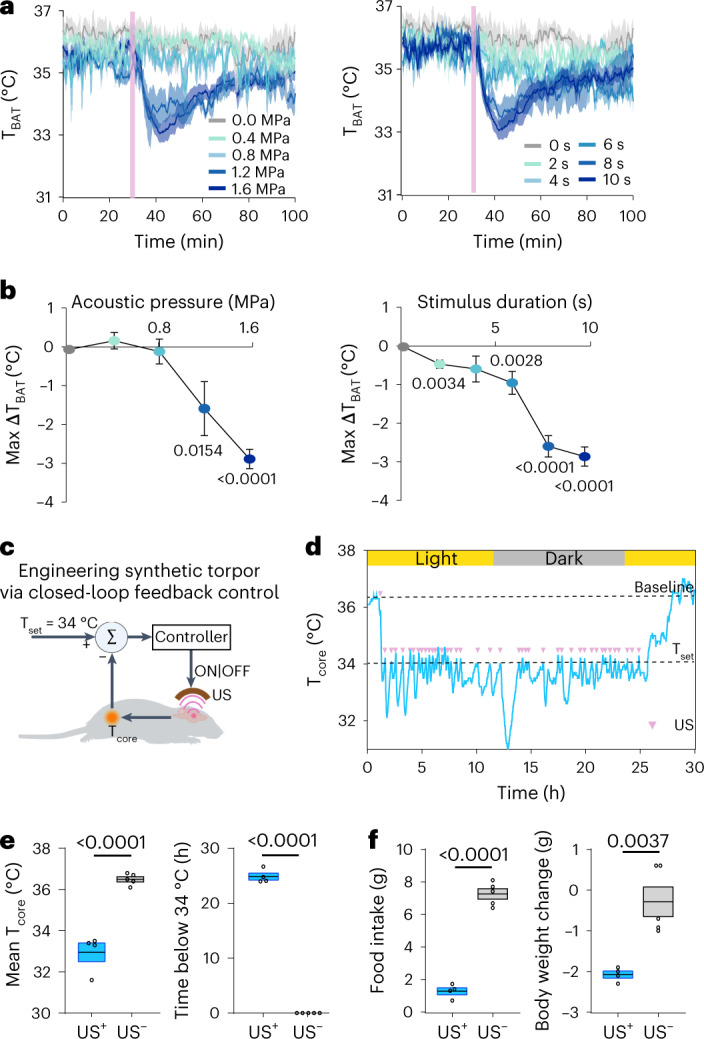
Precise control of the depth and duration of ultrasound-induced hypothermia. **a**, T_BAT_ measured with US stimulation at different acoustic pressure (left) and stimulus duration (right). Solid lines and shadows in curves denote the mean ± s.e.m. **b**, Correlations between max ΔT_BAT_ (lowest T_BAT_ − mean T_BAT_ before US) and acoustic pressure (left) or stimulus duration (right). *n* = 4 mice for the 0.4-MPa, 1.2-MPa, 2-s, 4-s and 6-s groups; *n* = 7 for the 0-MPa and 0-s groups; *n* = 3 for the 0.8-MPa and 8-s groups and *n* = 6 for the 1.6 MPa and 10-s groups). Error bars denote s.e.m. **c**, Schematic illustration of the closed-loop feedback control system for achieving long-duration UIH (created with BioRender.com). **d**, Representative T_core_ measured in one mouse with the closed-loop feedback-controlled UIH. Each US stimulus is represented by a pink dot. **e**, Quantification of the mean T_core_ (left) and duration when T_core_ < 34 °C (right) achieved by the closed-loop feedback control system. **f**, Food intake (left) and body weight change (right) of the mice that underwent closed-loop feedback-controlled UIH (US^+^, *n* = 4 mice), compared to the control mice (US^−^, *n* = 4 mice). For the box plots, the center line and box boundaries indicate mean ± s.e.m. *P* values were calculated using a two-tailed unpaired *t*-test. Source data

We next investigated the capability of ultrasound to precisely control the depth and duration of hypothermia. This capability to precisely control the induced state is the key to successfully engineering the torpor-like state. The effect of ultrasound on the brain depends on many parameters, including acoustic pressure and stimulus duration. We observed that higher acoustic pressure and stimulus duration increased the depth of UIH, as measured by max ∆T_BAT_ (Fig. 3a,b). An acoustic pressure higher than 0.8 MPa was needed to cause a significant change in ∆T_BAT_ under the parameter space tested in this study. We developed an automatic closed-loop feedback controller to achieve long-duration and stable UIH by binary control of the ultrasound output. As a proof of concept, the closed-loop feedback controller set the desired body temperature (T_set_) to be lower than 34 °C, which was previously reported as a criterion for natural torpor in mice^27^ (Fig. 3c). The controller continuously received input signals from the core body temperature sensor and then turned on the ultrasound stimulation when T_core_ > T_set_ or off when T_core_ ≤ T_set_. Core body temperature was recorded for a total duration of 30 h and the feedback-controlled ultrasound procedure continued for 24 h. The results showed that the feedback-controlled UIH maintained the mouse body temperature at 32.95 ± 0.45 °C for approximately 24 h (24.90 ± 0.63 h) (Fig. 3d,e). Mice showed reduced food intake and weight loss with feedback-controlled UIH compared to controls (Fig. 3f). The mouse’s body temperature gradually recovered to a normal level (>34 °C) at 54.18 ± 35.56 min after the feedback-controlled UIH ended (Extended Data Fig. 2e). The closed-loop feedback control system reveals the great potential of ultrasound-brain interfacing technology for noninvasive, precise induction of UIH.

### UIH is evoked by ultrasonic activation of POA neurons

**Fig. 4:**
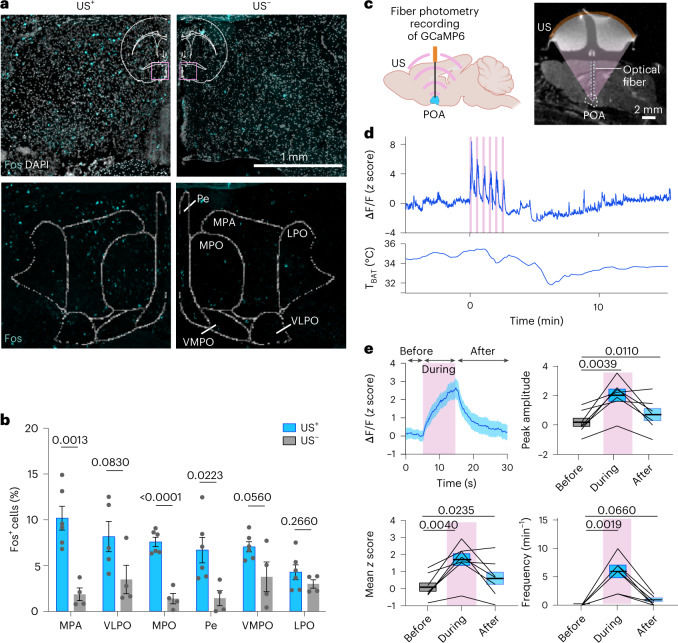
Ultrasound induces hypothermia and hypometabolism by activating neurons in the POA. **a**, Top: In situ hybridization analysis of the *Fos* marker in the POA region in mice with (US^+^) and without (US^–^) US stimulation. The insert shows the brain region where these images were obtained. Spatial distribution of the *Fos* signal registered with the mouse brain atlas (bottom). LPO, lateral preoptic area; VLPO, ventrolateral preoptic nucleus; VMPO, ventromedial preoptic nucleus. **b**, Fraction of *Fos*^+^ cells in different POA brain regions in the US^+^ and US^–^ groups. *n* = 6 mice for the US^+^ group and *n* = 4 mice for the US^-^ group, with the exception of the VLPO quantification in the US^+^ group where *n* = 5 mice were used. Error bars denote s.e.m. **c**, Schematic illustration for fiber photometry recording of neuronal Ca^2+^ activity in the POA targeted by US (created with BioRender.com) (left). MRI of a mouse shows the confocal alignment of optical fiber (green dotted line) and US transducer (brown) at the POA region (right). **d**, Representative Ca^2+^ activities (top) of POA neurons receiving US stimulation and the corresponding T_BAT_ curve (bottom). US stimulation is composed of six individual stimuli. **e**, Ca^2+^ signal in response to each US pulse from *n* = 7 mice. Based on this Ca^2+^ signal, the peak amplitude, mean *z* score and frequency were quantified in three windows before (5 s), during (10 s) and after (15 s) US stimulation (*n* = 7 mice). For the box plots, the center line and box boundaries indicate mean ± s.e.m. *P* values were calculated by the unpaired two-tailed *t*-test (**b**) and paired two-tailed *t*-test (**e**). Source data

**Extended Data Fig. 6:**
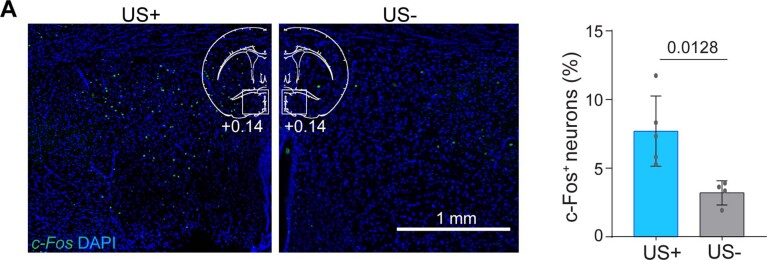
C-Fos expression at POA induced by ultrasound stimulation. Left: Immunofluorescence staining of c-Fos protein in the ultrasound-stimulated brain (US+) and non-sonicated brain (US^−^). Right: Percentage of c-Fos^+^ cells among all the cells in the targeted POA region when comparing US+ and US^−^ groups. N = 5 mice for the US^+^ group and 4 mice for the US^−^ group. Error bars denote s.e.m. P values were calculated using unpaired two-tailed t-tests. Source data

To understand the neuronal mechanism of UIH, we started with ex vivo fluorescence in situ hybridization (FISH) and immunofluorescence staining of the neuronal activity marker *Fos* at the ultrasound-targeted POA region (Fig. 4a and Extended Data Fig. 6). The *Fos* expression was high in the medial preoptic area (MPA; 10.17 ± 1.30% versus 1.85 ± 0.69%), medial preoptic nucleus (MPO; 7.58 ± 0.51% versus 1.38 ± 0.57%) and periventricular nucleus (Pe; 6.68 ± 1.39% versus 1.43 ± 0.82%) (Fig. 4b). We then studied the dynamics of neuronal activity in the POA in response to ultrasound. We expressed the calcium (Ca^2+^) reporter GCaMP6s in POA neurons, installed an optical fiber to the same region and continuously recorded the dynamics of Ca^2+^ activity before, during and after ultrasound stimulation using fiber photometry (Fig. 4c). We observed a consistent, robust and repeated increase in neuronal activity in response to each ultrasound pulse (Fig. 4d). The recorded neuronal activity during ultrasound stimulation was characterized by a rise in the peak amplitude (from 0.17 ± 0.30 to 2.03 ± 0.42), mean *z* score (from 0.09 ± 0.28 to 1.69 ± 0.40) and peak frequency (from 0 to 5.7 ± 1.08 min^−1^) compared to neuronal activity measured before ultrasound stimulation (Fig. 4e). The trend of Ca^2+^ activity changes was aligned with the trend of body temperature changes (Fig. 4d). These findings revealed that UIH was evoked by ultrasound activation of POA neurons. Our finding that transcranial stimulation of the POA was sufficient to induce UIH revealed the critical role of the POA in orchestrating a torpor-like state in mice.

### TRPM2 facilitates ultrasonic activation of POA^UIH^ neurons

**Fig. 5:**
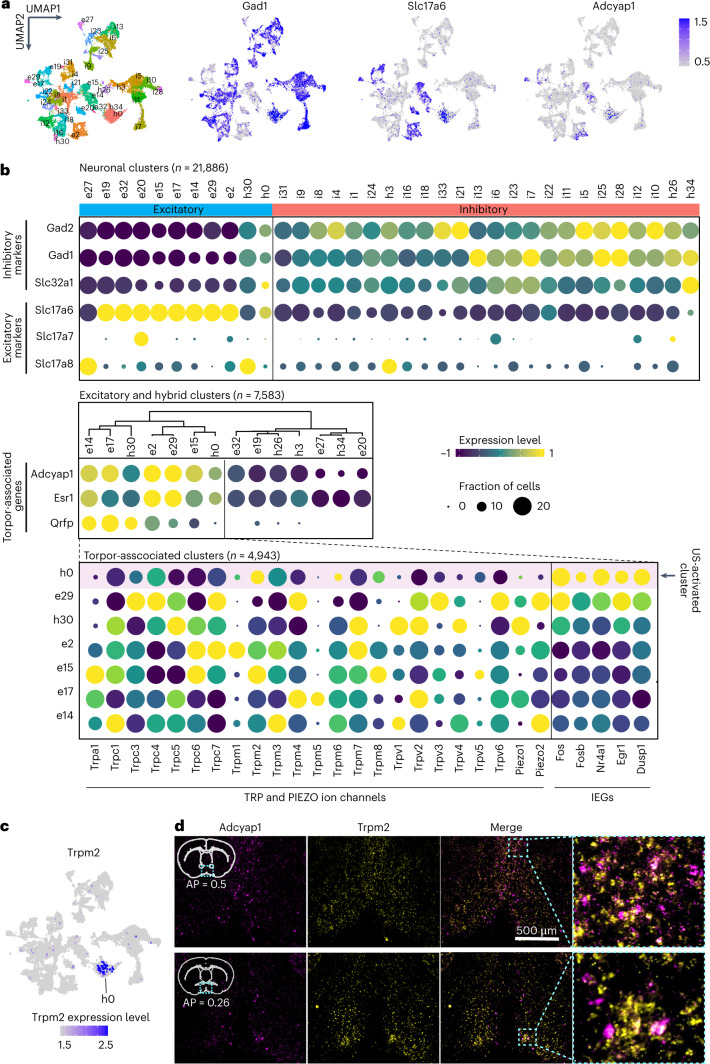
Molecular characterization of POA^UIH^ neurons. **a**, Uniform Manifold Approximation and Projection (UMAP) plot of 21,886 neuronal nuclei. Different colors represent different neuronal populations. Expression levels of three main representative class-specific markers genes are color coded (blue) on UMAP: *Gad1* for inhibitory clusters, *Slc17a6* for excitatory clusters and *Adcyap1*^+^ for torpor-associated clusters. **b**, Expression of excitatory and inhibitory marker genes across different neuronal cell types (organized by *k*-means clustering) (top). Expression of torpor-associated marker genes (*Adcyap1*, *Esr1* and *Qrfp*) across excitatory and hybrid neuronal populations (clustered based on these torpor-associated genes) (middle). Expression of all the genes encoding TRP and PIEZO channel families across all the torpor-associated clusters, which were ranked by the expression level of IEGs (bottom). The pink bar indicates the cluster that was activated by US. **c**, The expression level of *Trpm2* is color coded (blue) on UMP. US-activated torpor-associated h0 cluster is labeled in the plot. **d**, Representative FISH image of *Adcyap1* and *Trpm2* in POA region of two coronal sections from different levels. FISH analyses were repeated in five mice. Anterior–posterior coordinates relative to bregma are indicated based on the Allen Mouse Brain Atlas^62^.

To identify the ultrasound-sensitive molecule that enables the activation of POA neurons associated with the induction of UIH (POA^UIH^ neurons), we performed a high-throughput single-nucleus RNA-sequencing (snRNA-seq) of the ultrasound-targeted POA region. We dissected the POA regions (*n* = 4 mice for the US^+^ group and *n* = 4 mice for the US^−^ sham group), dissociated them to collect single nuclei and pre-select nuclei from neurons using the NeuN marker. A total of 35 diverse neuronal clusters containing 21,886 neurons (Fig. 5a) were identified using unsupervised graph-based clustering^28^. Among them, 11 clusters are excitatory (including 2 hybrid clusters) and 24 are inhibitory (including 3 hybrid clusters) (Fig. 5b), which is consistent with the high diversity of cell types in the POA^11,29^. Recent studies identified that torpor-associated neurons are characterized by specific genetic markers, including *Adcyap1* (encoding adenylate cyclase activating polypeptide-1)^11^, *Qrfp* (pyroglutamylated RFamide peptide)^12^ and *Esr1* (estrogen receptor-1)^15^. Additionally, a majority of these neurons were classified as either excitatory or hybrid. We used these three markers to identify torpor-associated neurons by performing *k*-means clustering^30^ and found seven torpor-associated clusters that were excitatory or hybrid neurons (Fig. 5b).

The commonly accepted hypothesis for ultrasound neuromodulation is the activation of endogenous ultrasound-sensitive ion channels. Although previous studies reported the activation of several ion channels from PIEZO and TRP families (including PIEZO1/2 (refs. ^31,32^), TRPA1 (ref. ^33^), TRPV1 (ref. ^34^), TRPP1/2 and TRPC1 (ref. ^35^)), these studies were performed mainly in vitro using cultured cells. No study has been performed in vivo to screen candidate ion channels for revealing the ultrasound-sensitive ion channels on neurons in the brain. Here, we examined all the genes from TRP (*TRPV*, *TRPM*, *TRPC*, *TRPP*, *TRPA* and *TRPML*) and PIEZO channel families in the torpor-associated excitatory neurons and found broad expression of genes encoding TRP channel families, including TRPV, TRPM, TRPC, TRPA and PIEZO ion channels (Fig. 5b). Among all seven torpor-associated neuronal populations, we then identified a specific population that was characterized by high expression of five neuronal activation markers, including *Fos*, *Fosb*, *Nr4a1*, *Egr1* and *Dusp1*, also known as immediate-early genes (IEGs), indicating that this neuronal population was activated by ultrasound. We refer to the ultrasound-activated torpor-associated neuronal population POA^UIH^ neurons. By assessing all TRP and PIEZO channel genes, we found that the expression level of TRPM2 was high in the POA^UIH^ neurons (Fig. 5b,c).

**Fig. 6:**
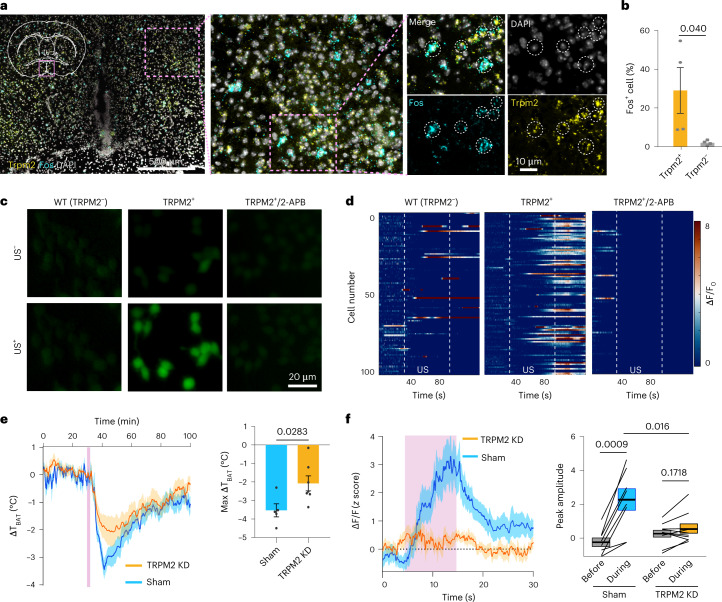
TRPM2 is an ultrasound-sensitive ion channel and involved in the induction of UIH. **a**, Representative FISH images of *Fos* and *Trpm2*. Cells coexpressing *Fos* and *Trpm2* are circled. **b**, The percentage of *Fos*^+^ cells in populations expressing *Trpm2* (*Trpm2*^+^) compared to the population lacking expression of *Trpm2* (*Trpm2*^−^). *n* = 4 mice. **c**, Fluorescence Ca^2+^ images of in vitro HEK293T cells without TRPM2 overexpression (left), with TRPM2 overexpression (middle) and with TRPM2 overexpression and 2-APB blocker (right). Before US stimulation (top); after US stimulation (bottom). *n* = 5 independent tests. WT, wild type. **d**, Heat map of normalized Ca^2+^ signal (ΔF/F) of 100 randomly selected cells from five independent tests in these three groups. Dotted lines indicate the on and off time of US stimulation. **e**, ΔT_BAT_ (change of the T_BAT_ relative to the baseline) of mice injected with shRNA to knockdown TRPM2 (TRPM2-KD) (left). Max ΔT_BAT_ (right) was determined as the lowest ΔT_BAT_ during the 7–12-min interval after the onset of US stimulation, based on the left plot. The sham group received an injection of scrambled shRNA. *n* = 5 mice for the sham group and *n* = 7 mice for the TRPM2-KD group. **f**, Ca^2+^ signal in response to US sonication (pink bar) in TRPM2-KD mice compared to the sham group. The peak amplitude of the Ca^2+^ curve was quantified for the TRPM2-KD group and the sham group. *n* = 8 mice for the sham group and *n* = 10 mice for the TRPM2-KD group. Solid lines and shadows in curves denote the mean ± s.e.m. Error bars denote s.e.m. US stimulation is marked by pink bars. *P* values were calculated using unpaired two-tailed *t*-test in **b** and **e**. Paired two-tailed *t*-test was used in **f** when comparing the peak amplitude before and during US stimulation. Source data

To verify that the TRPM2 ion channel is sensitive to ultrasound, we performed ex vivo FISH analysis and found that *Trpm2* was coexpressed with *Fos*, with a coexpression percentage of 27.53 ± 9.75% (Fig. 6a,b). We then further evaluated the ultrasound sensitivity of TRPM2 by overexpressing it in HEK293T cells. We found that ultrasound successfully induced Ca^2+^ influx in cells overexpressing TRPM2, which was not observed in the wild-type cells (Fig. 6c,d). The ultrasound-evoked Ca^2+^ response in TRPM2^+^ cells was significantly suppressed by the TRPM2 blocker 2-APB (Fig. 6c,d). These findings strongly support that TRPM2 is an ultrasound-sensitive ion channel.

**Extended Data Fig. 7:**
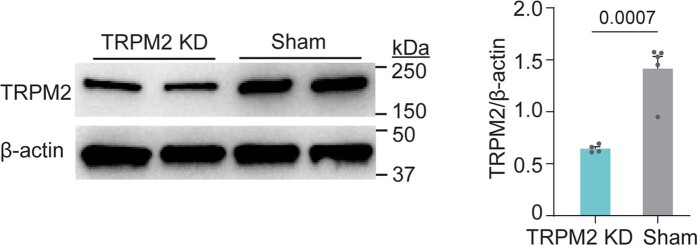
Validation of TRPM2 knockdown at POA. Western blot evaluation of the TRPM2 expression level in sham mice receiving the injection of control virus (Sham) and mice receiving Lentivirus-Sh-RNA injection to knockdown TRPM2 expression (TRPM2-KD). Error bars denote s.e.m. N = 4 mice for the TRPM2-KD group and 5 mice for the Sham group. P values were calculated using unpaired two-tailed t-tests. Source data

To verify that the TRPM2 ion channel is involved in the UIH, we performed in situ hybridization analysis and found that *Trpm2* was colocalized with the molecular marker of torpor-associated neurons *Adcyap1* with a coexpression percentage of 35.7 ± 5.3% (Fig. 5d). We further investigated the roles of TRPM2 in UIH by using lentivirus-shRNA to specifically knock down TRPM2 expression levels (TRPM2-KD) at the POA region. Western blot analysis confirmed the successful knockdown of TRPM2 (Extended Data Fig. 7). TRPM2-KD mice significantly diminished UIH. The T_BAT_ temperature reduction was decreased by 41.2% in TRPM2-KD mice compared to the control (Fig. 6e). The amplitude of ultrasound-evoked Ca^2+^ signal in POA decreased to almost baseline in TRPM2-KD mice (Fig. 6f). These findings demonstrated that TRPM2 was involved in the induction of UIH.

### Downstream brain regions and effector tissue involve in UIH

**Fig. 7:**
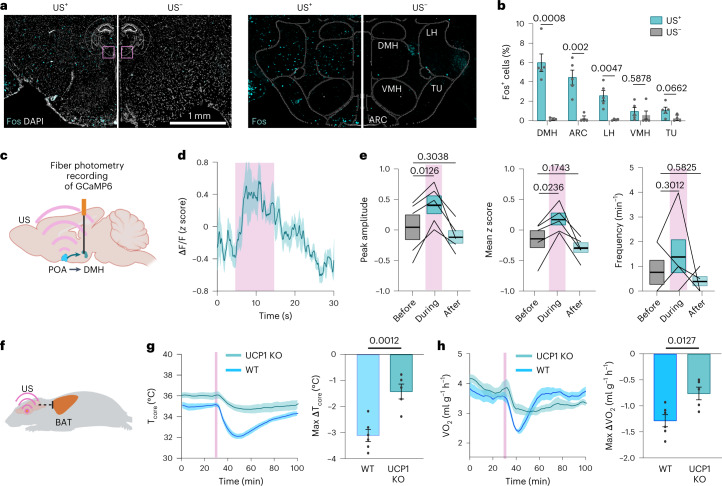
UIH is associated with DMH activation and BAT regulation. **a**, FISH staining of *Fos* marker in hypothalamic regions around DMH in mice with (US^+^) and without (US^–^) US stimulation (left). The locations of these images are indicated in the brain atlas (insert). Spatial distribution of the *Fos* signal registered in mouse brain atlas (right). TU, olfactory tubercle. **b**, Fraction of *Fos*^+^ cells in the DMH, ARC, LH, VMH and TU for US^+^ and US^–^ mice. *n* = 5 mice for the US^+^ group and *n* = 4 mice for the US^−^ group. Error bars denote s.e.m. **c**, Setup for photometry recording of neuronal Ca^2+^ activity in the DMH simultaneously with US targeting at the POA. **d**, Ca^2+^ activity of DMH neurons during US stimulating POA neurons. *n* = 5 mice. Solid lines and shadows in curves denote the mean ± s.e.m. **e**, Peak amplitude (left), mean *z* score (middle) and frequency (right) of DMH neuronal activity before, during and after US stimulation at the POA region. *n* = 5 mice. **f**, Illustrative of the hypothesis that UIH is mediated by UCP1-dependent BAT thermogenesis and energy expenditure. **g**, T_core_ during US stimulation comparing WT and UCP1-knockout (UCP1-KO) mice (left). Max ΔT_core_ (lowest T_core_ – mean T_core_ before US) (right). **h**, VO_2_ during US stimulation in WT and UCP1-KO mice (left). Max ΔVO_2_ (lowest VO_2_ – mean VO_2_ before ultrasound stimulation) (right). In **g** and **h**, solid lines and shadows in curves denote the mean ± s.e.m.; error bars denote s.e.m.; *n* = 6 mice for the WT group and *n* = 5 mice for the UCP1-KO group. *P* value was calculated using the unpaired two-tailed *t*-test (**b**,**g**,**h**) and paired two-tailed *t*-test (**e**). **c** and **f** were created with BioRender.com. Source data

We then studied the potential downstream brain regions associated with the activation of POA neurons during UIH. Metabolic and thermal regulations were previously reported to be coordinated by POA projections to the dorsomedial hypothalamus (DMH)^36,37^, arcuate nucleus (ARC)^36^ and ventromedial hypothalamus (VMH)^36^, which are located in the hypothalamic region posterior to the POA. We performed the FISH analysis of the *Fos* marker in these hypothalamic regions following ultrasound stimulation at the POA. We detected increased *Fos* expression in multiple hypothalamic areas, including DMH, ARC and the lateral hypothalamus (LH) (Fig. 7a,b). The highest *Fos* expression was observed in DMH (6.00 ± 0.91% in US^+^) (Fig. 7b).We recorded the Ca^2+^ signal in the DMH region using fiber photometry while simultaneously targeting ultrasound at the POA to study the DMH neural activity during UIH (Fig. 7c). We observed that the mean peak amplitude of the Ca^2+^ signal of DMH neurons increased during ultrasound stimulation at the POA (from 0.04 ± 0.20 to 0.41 ± 0.15; Fig. 7d,e), demonstrating that the activation of DMH neurons is associated with the UIH. These results indicated that DMH is potentially involved in the neural circuit that regulates UIH, while other brain regions (for example, ARC and VMH) may also act as nodes in the torpor neural network.

The hypothalamus is known to be connected with the sympathetic nervous system that alters thermogenesis and energy expenditure of peripheral effector tissues^38^. We observed the decrease of BAT temperature during UIH (Fig. 2a,b), indicating that BAT is a downstream effector tissue of UIH neural circuits (Fig. 7f). As UCP1 is the crucial mitochondrial protein responsible for BAT thermogenesis^39^, we used UCP1-KO mice for UIH and compared the changes in T_core_ and VO_2_ with those of the wild-type control. We found that the depth of ultrasound-induced hypothermia (measured by max ΔT_core_) was ~2.2-fold lower in UCP1-KO mice than in wild-type mice (Fig. 7g). The extent of hypometabolism (measured by max ΔVO_2_) was 1.7-fold lower in UCP1-KO mice than in wild-type mice (Fig. 7h); however, this hypothermic and hypometabolic state was not entirely abolished in UCP1-KO mice. These findings confirmed that BAT is one effector tissue that mediates the body temperature and metabolic rate changes during UIH.

### Ultrasound induces hypothermia in a non-torpid animal: rat

**Fig. 8:**
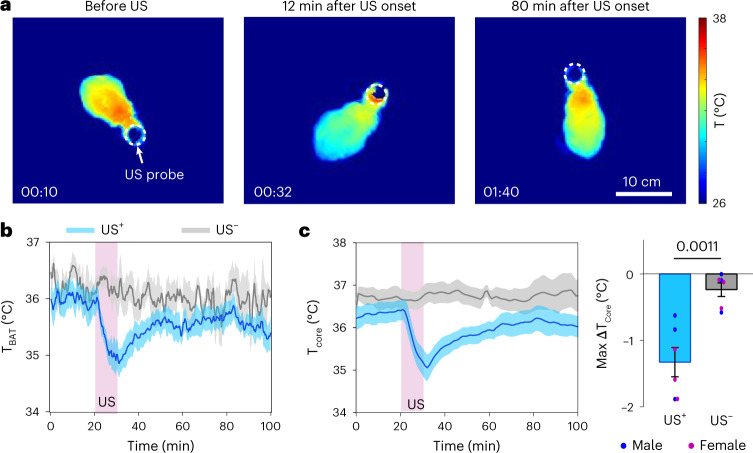
Ultrasound stimulation at the POA induces hypothermia in rats. **a**, Infrared thermal images of a rat receiving US stimulation at the POA. **b**, T_BAT_ of rats receiving sonication at the POA (US^+^, *n* = 12) compared to the control rats without US stimulation (US^–^, *n* = 4). **c**, T_core_ curves in US^+^ (*n* = 6) and US^–^ (*n* = 6) groups (left). Comparison of max ΔT_core_ between the US^+^ and US^–^ groups (right). Solid lines and shadows in curves denote the mean ± s.e.m. Error bars denote s.e.m. The *P* value was calculated using a two-tailed unpaired *t*-test. Source data

Mice are well equipped to enter a state of torpor under caloric restriction. To evaluate whether UIH can be evoked in non-torpid animals, we performed ultrasound stimulation in rats, a species that shows neither hibernation nor daily torpor^12,14^. Ultrasound was remotely delivered to the POA region of freely moving rats using the wearable ultrasound transducer with a similar design as that used in mice (Fig. 1b). We found a decrease in skin temperature, particularly in the BAT (T_BAT_) region after ultrasound stimulation (Fig. 8a,b). Ultrasound stimulation also induced a significant decrease in core body temperature (max ∆T_core_ = −1.33 ± 0.22 °C) in rats (Fig. 8c). Though the degree of body temperature reduction was smaller in rats than mice, these data demonstrate the feasibility of UIH in a non-torpid animal.

## Discussion

In this study, we developed the UIH technique that noninvasively, precisely and safely induced a torpor-like state in mice. We revealed that UIH was evoked by ultrasound activation of POA neurons. POA^UIH^ neurons had high expression of the TRPM2 ion channel, which was found to be ultrasound sensitive and contributed to the induction of UIH. Additionally, our findings indicate the potential involvement of the dorsomedial hypothalamus as a downstream brain region and brown adipose tissue as an effector tissue in UIH. We demonstrated that ultrasound stimulation at POA evoked a hypothermia state in a non-torpid animal (rat).

**Extended Data Fig. 8:**
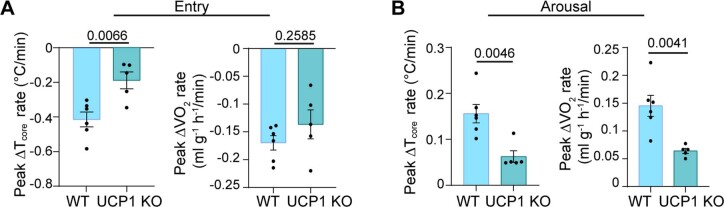
Characterization of the entry and arousal pattens during UIH in WT and UCP1-KO mice. Quantification of the body temperature change rate (peak ΔT_core_ rate) and VO_2_ change rate (peak ΔVO_2_ rate) during the entry (**A**) and arousal of UIH (**B**) for WT and UCP1-KO groups. Error bars denote s.e.m. Each dot represents one mouse (female). N = 6 mice for the WT group and 5 mice for the UCP1-KO group. P values were calculated using unpaired two-tailed t-tests. Source data

Our findings demonstrated that ultrasound stimulation of the POA globally suppressed physiological processes in a manner that resembled natural torpor. Ultrasound stimulation systemically suppressed metabolism and thermogenesis, switched energy substrate utilization to fat and decreased heart rate, which collectively are key features of natural torpor^11,23^. Single ultrasound stimulation was found to induce a hypothermic and hypometabolic state lasting up to approximately 1 h, which is consistent with natural torpor^24,25^. This prolonged duration was also consistent with a previous report that found optogenetic activation of QRFP neurons in the POA resulted in a state of hypothermia lasting for longer than 30 min following a 1-s light illumination^12^. The relatively prolonged duration of this state is partially attributed to the slow process of thermogenesis during rewarming^40^. It could also be possible that neural circuits involved in maintaining this hypothermic and hypometabolic state may be activated by ultrasound stimulation directly or indirectly. Despite the similarity between UIH and natural torpor, there are still differences between these two states regarding the downstream effector tissues. We found that BAT is involved in the UIH process mediated by UCP1 that regulates both the depth of hypothermia and hypometabolism and the arousal from UIH (Extended Data Fig. 8). In natural torpor, however, UCP1 was reported to mainly contribute to the arousal from torpor, not the depth of hypothermia and hypometabolism^40^. In addition, mouse tail temperature increased during UIH, possibly to enhance thermal dissipation via vasodilation. This phenomenon was not reported in fasting-induced daily torpor^41^. Additional research is necessary to characterize and understand the similarities and differences between the UIH state and natural torpor with regard to physiological features, neural circuits and effector tissues.

The globally coordinated regulation of torpor-associated behaviors infers that ultrasound modulated the torpor-associated neuronal populations in POA. Indeed, the single-nucleus RNA-sequencing revealed that ultrasound activated the previously reported torpor-associated neuronal populations in POA, which are characterized by the marker genes of *Adcyap1*, *Qrfp* and *Esr1* (refs. ^11,12,15^). We discovered that TRPM2 is an ultrasound-sensitive ion channel in POA^UIH^ neurons. Our study provides in vivo evidence revealing that ultrasound modulates neuronal activities in the brain by activating endogenous ion channels. TRPM2 was previously discovered in warm-sensitive POA neurons that regulate body temperature^42^. We found that TRPM2 is necessary for inducing UIH. This study focused on TRPM2, which was the highest expressed ion channel in POA^UIH^ neurons among those ion channels included in our analysis. In addition to TRPM2, we also observed broad expression of other TRP and PIEZO channel families in torpor-associated neuronal populations. TRPM2 knockdown partially suppressed the depth of UIH but did not entirely abolish the UIH effect, suggesting that other ultrasound-sensitive ion channels or molecules are also involved in the ultrasonic activation of POA neurons to induce hypothermia and hypometabolism.

**Extended Data Fig. 9:**
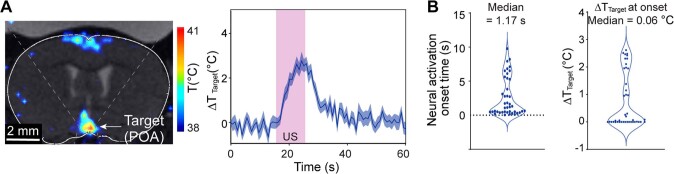
Measurements of the ultrasound-induced thermal effect in vivo. (**A**) (Left) Coronal view MR thermometry image of ultrasound-induced heating in the mouse brain in vivo. The solid white line outlines the mouse brain, and the dotted lines illustrate the ultrasound beam. (Right) Temperature changes within the ultrasound-targeted POA region (ΔT_Target_) as a function of time. (**B**) (Left) Neural activation onset time was calculated from the fiber photometry recording of Ca^2+^ activity at the POA during US stimulation (Fig. 4). (Right) The corresponding ΔT_Target_ at the onset time of neural activation. Solid lines and shadows denote the mean ± s.e.m. Each dot represents one 10-s ultrasound stimulation. Source data

**Extended Data Fig. 10:**
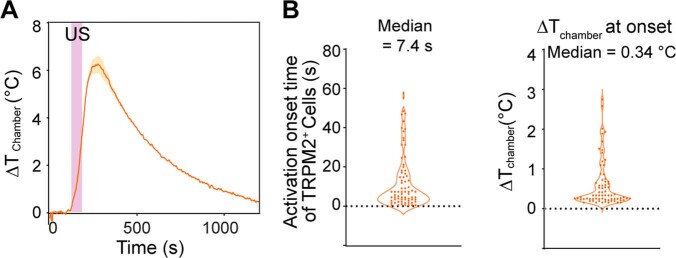
Measurements of the ultrasound-induced thermal effect in vitro. (**A**) Temperature changes in the cell culture chamber (ΔT_chamber_) as a function of time. (**B**) (Left) The activation onset time of TRPM2^+^ cells was calculated from the fluorescence Ca^2+^ images of in vitro HEK293 cells with TRPM2 overexpression (Fig. 6). (Right) The corresponding ΔT_chamber_ at the onset time of TRPM2 activation. Solid lines and shadows denote the mean ± s.e.m. Each dot represents one cell. Source data

The biophysical mechanisms for ultrasound activation of POA neurons and the TRPM2 ion channel remain to be discovered. TRPM2 is a Ca^2+^-permeable, non-selective cation channel and sensitive to temperature changes^43,44^. We measured the POA temperature during ultrasound sonication using in vivo magnetic resonance imaging (MRI) thermometry. The temperature rise at POA was 2.58 ± 0.26 °C (Extended Data Fig. 9), suggesting that the ultrasound-induced thermal effect contributed to the activation of POA neurons. By analyzing the correlation of neural activation and POA temperature rise, we found that the median onset time of neural activation occurred at 1.17 s. The median temperature rise at the onset time of neural activation was 0.06 °C (Extended Data Fig. 9). Such low temperature rise suggests that the ultrasound-induced mechanical (non-thermal) effect also contributed to the activation of the POA neurons. Similarly, we observed a temperature rise in the cell culture chamber during ultrasound stimulation of TRPM2^+^ HEK293 cells. We found that the median temperature rise corresponding to the onset of Ca^2+^ activity of TRPM2^+^ cells was 0.34 °C (Extended Data Fig. 10). In summary, the POA neurons and TRPM2 ion channel are likely to be activated by the mechanical and thermal effects of ultrasound.

POA neurons coordinate the activation of several downstream brain areas to orchestrate torpor; however, this brain-wide complex circuit is still largely unknown. We found that the induction of UIH was correlated with the activation of DMH neurons during ultrasonic stimulation at the POA. This finding is consistent with the previously reported neural circuits that both the *Qrfp* and *Adcyap1* torpor-associated POA neurons project to DMH to induce hypothermia and hypometabolism during genetically induced or fasting-induced torpor^11,12^. Our study also suggests that other downstream brain regions, such as the ARC, may also be involved in the coordination of UIH. The ARC, an area in the hypothalamus that is critical for the regulation of energy homeostasis, was suggested to be the crucial downstream region receiving the projection from torpor-inducing *Esr1*^+^ neurons in POA^15^. These findings suggest that ultrasound stimulation induces torpor-like hypothermia and hypometabolism by modulating the neural circuits involved in natural torpor. This study also found that the downstream effector tissue, BAT, was involved in the UIH regulation. BAT was previously studied in the context of thermogenesis^39,45^, whereas its role in torpor regulation was not fully explored^40,46,47^. We found that BAT is one of the key effector tissues that mediate the body temperature and metabolic rate changes during UIH and that these effects are UCP1-dependent.

UIH has the potential to address the long sought-after goal of achieving noninvasive and safe induction of the torpor-like state, which has been pursued by the scientific community at least since the 1960s. In the past, extensive efforts were devoted to identifying pharmacological agents that systemically suppress metabolism and induce a torpor-like state. These agents, such as hydrogen sulfide^9^, adenosine monophosphate^48^, delta-opioids^49^, N6-cyclohexyladenosine^14,50^ and the cocktails of chemical compounds^51^, lack anatomical and functional specificity and induce systemic off-target side-effects^52^, all of which limit their uses. Recent studies proposed approaches to induce torpor by targeting the CNS through surgical injection of pharmaceutical agents into the brain or genetic engineering of torpor-associated neural circuits. These approaches are critically important to investigate the central nervous pathway of torpor, but the needs for surgical intervention in the brain and genetic engineering limit their translation to humans. Compared to other non-pharmacological and non-genetic neuromodulation techniques (for example, deep brain stimulation, transcranial magnetic stimulation and transcranial direct current stimulation), ultrasound stimulation possesses a unique capability to noninvasively reach deep brain regions with high spatial and temporal precision in animal and human brains. UIH utilizes transcranial ultrasound stimulation to induce hypothermia and hypometabolism by noninvasively stimulating the master regulator of torpor, the POA. It represents a noninvasive and safe technique that induces torpor-like hypothermia and hypometabolism by targeting the CNS.

As ultrasound neuromodulation has already been demonstrated to be feasible in humans, UIH has excellent promise for translating to humans. A valid question is whether UIH can be extrapolated from mice to humans. We showed that ultrasound stimulation induced hypothermia in rats, which do not naturally enter torpor, suggesting the possibility that similar effects could be induced in humans. Precise ultrasound regulation of POA neurons may achieve torpor-like hypothermia and hypometabolism in humans, although much work needs to be conducted. UIH may unlock applications ranging from new medical treatments to long-duration human spaceflight.

## Methods

### Animals

For the initial characterization of the US-induced torpor-like hypothermia and hypometabolism effect, we used adult (6–9 weeks old) male and female C57BL/6NCrl mice (Charles River Laboratories) that were randomly assigned to experimental groups. UCP1-knockout mice (female, 2–4 months old) were provided by J. Brestoff’s laboratory. Female and male Wistar Han IGS rats (Charles River Laboratories, strain code 273) at the age of 4–6 weeks were used in this study. All mice and rats were housed in an animal facility at Washington University School of Medicine. Animals were maintained in a temperature-controlled (23–26 °C) and humidity-controlled (35–65%) environment, with a 12-h light–dark cycle and provided with a standard chow diet. All animal studies were reviewed and approved by the Institutional Animal Care and Use Committee of Washington University in St. Louis in accordance with the National Institutes of Health (NIH) Guidelines for Animal Research (animal protocol no. 21-0187).

### Ultrasound stimulation

We designed miniaturized wearable US transducers to deliver US stimulation in freely moving mice. The transducer was made using a lead zirconate titanate ceramic resonator (DL-43, DeL Piezo Specialties) with a center frequency of 3.2 MHz, an aperture of 13 mm and a geometric focal depth of 10 mm. Every transducer was calibrated by a hydrophone (HGL-200, Onda) in degassed and deionized water with and without an ex vivo mouse skull. The full width at half maximum of the US transducer was 0.8 mm and 3.8 mm in the lateral and axial directions, respectively.

The wearable US transducer was attached to the mouse head by the ‘plug-in’ design onto a baseplate that was glued on the mouse skull before the experiment (Extended Data Fig. 1). To glue the baseplate, the mouse was anesthetized, and the head was fixed using a stereotactic frame. A marker pen was attached to the stereotactic frame and used to draw a dot on the skull to indicate the POA region using its coordinates in reference to the bregma (AP = 0.2 mm and ML = 0.2 mm relative to the bregma). The baseplate was implanted onto the top of the mouse’s skull with the aid of an insert. The insert had a hole in the center, which was aligned with the dot on the skull to target the desired brain region. After this targeting process, the insert was removed, and the baseplate was glued on the skull using adhesive cement. For the off-target control experiment, the baseplate was implanted to target the cortex (AP = 3.0 mm and ML = 0 mm, relative to bregma) or the ventral hippocampus, which is located at the same depth as POA (AP = −2.7 mm, ML = 2.7 mm and DV = −5.0 mm). After baseplate implantation, the animals were allowed to recover from surgery and adapt to the baseplate for at least 3 d. Before each US stimulation experiment, the wearable US transducer was filled with degassed US gel (Aquasonics) and then plugged into the baseplate on the mouse head under light anesthesia using isoflurane (1–1.5% isoflurane). The total duration of this preparation was at most 15 min to minimize the effect of anesthesia. The US targeting depth (DV direction) was controlled by customizing the height of the cone-shaped housing. The focal length of the concave-shaped transducer was ~8 mm. The height of the transducer housing was designed to be approximately 3 mm to target the POA and ventral hippocampus (as an off-target control), which are at a depth of 5 mm. The height of the transducer housing was designed to be 7 mm to target the cortex, which is at a depth of 1 mm. The focal depth (relevant to the skull) of the wearable US was validated by the hydrophone calibration with the ex vivo mouse skull (Extended Data Fig. 1). Mice were then allowed to recover from anesthesia and habituate to the behavior testing platform for approximately 90 min before the first stimulation. The baseplate installation and the wearable US transducer design were similar in the rat experiments, except for the center frequency (1.5 MHz in rat experiments) and the targeting coordinates (AP = 0 mm, ML = 0 mm and DV = ~8.5 mm).

US stimulation was performed using the following parameters: fundamental frequency of 3.2 MHz for mice and 1.5 MHz for rats, peak negative acoustic pressure (in situ pressure with skull attenuation considered) of 1.6 MPa for mice and 1.4 MPa for rats, duty cycle of 50%, pulse repetition frequency of 10 Hz, stimulus duration of 10 s, inter-stimulus interval of 30 s, stimulus number of 6 (mice) or 20 (rats). In the parameter study, the stimulus duration (2–10 s) and acoustic pressure (0.4, 0.8, 1.2 and 1.6 MPa) were varied, while other parameters were kept the same. To avoid activating the auditory pathway by US stimulation, we applied a smoothed envelope at the beginning and end of each US waveform using a previously reported method^53^. The waveform was generated in MATLAB (R2021a, Mathworks) by applying a gaussian function envelope at the beginning and end of the waveform to reduce the generation of broadband frequency components. The pulse repetition frequency was 10 Hz, which is outside of the mouse hearing range^54,55^. The programmed waveform was then input to a function generator (33500B, Agilent), amplified via a power amplifier (1020L, ENI) and transmitted to the US transducer. No electrical impedance matching was needed because the real part of the transducer impedance at the resonance frequency, as measured by an E5061A ENA Network Analyzer with the 85070E Dielectric Probe kit (Agilent Technologies), was in the range of 31 to 59 ohms, which was sufficiently close to the 50 ohms needed for a perfect impedance match.

### Body temperature and metabolism measurements

The surface body temperature before, during and after US stimulation was recorded using a thermal imaging camera. Mice with the wearable US transducer attached were put into an open-field testing platform, which was a box of 20 cm (height) × 10 cm (width) × 10 cm (length). An infrared thermal imaging camera (FLIR E54 camera, FLIR Systems) was positioned 60 cm above the box to capture the mouse’s body temperature with the FLIR ResearchIR (v.4) software. To ensure accurate surface temperature measurements, the fur on the animal’s back was removed using hair clippers before the start of the experiment. This hair removal procedure was performed in all experiments, including both the US^+^ and US^−^ control groups. A webcam (C920, Logitech) was placed next to the thermal camera to capture mouse behavior using Bonsai (v.2.7) software in synchronization with the thermal camera. The ambient temperature was kept consistent at ~22 °C for all the experiments. The T_BAT_ was automatically calculated using the combination of FLIR Tool (v.6.4), Bonsai and MATLAB software with the following steps: (1) tracking the BAT location in Bonsai by estimating the center of the BAT to be 25% of the body length anterior to the centroid of the body; and (2) calculating the mean temperature within the region of interest defined by a 3-mm-radius circle centered at the identified BAT location using MATLAB. The tail temperature was manually calculated by selecting a 2-mm-diameter circular region of interest centered at the proximal 1 cm of the tail. The maximum change in the BAT temperature (max ∆T_BAT_) was calculated by computing the difference between the minimum BAT temperature obtained within the 7–12 min interval following the start of US and the baseline body temperature, which was defined as the mean body temperature measured 15 min before the US stimulation. This calculation was carried out after applying a low-pass filter to the raw signal to eliminate high-frequency camera-induced fluctuation for a more accurate result.

For the measurements of core body temperature (T_core_), rate of oxygen consumption (VO_2_) and RQ recordings, each mouse was housed in a metabolic cage inside the temperature-controlled Comprehensive Laboratory Animal Monitoring System (CLAMS) (Columbus Instruments). The US transducer was attached to the top of the metabolic cage with a rotatory joint adaptor to ensure that the mouse was able to freely move in the cage with the wearable US transducer without the tangle of wire. Mice were initially adapted to the CLAMS for 1 d before every experiment. Oxygen consumption (VO_2_) and carbon dioxide production (VCO_2_) rates were measured using an OxyMax fast sensor (Columbus Instruments) every ~3 min with eight cages measured in series for 20 s each (18-s airline bleed; 2-s measurement) and recorded using CLAX Statistical Software. Mice in the metabolic cages were also implanted with telemetry temperature sensors (TA-XS, Stellar Telemetry, TSE Systems) for the recording of the core body temperature in synchronization with the metabolic recordings. Mice had ad libitum access to food and water throughout the experiment. The maximum change in the core body temperature (max ∆T_core_) was calculated by computing the difference between the minimum body temperature obtained within the 30 min period following the start of US and the baseline body temperature (defined as the mean body temperature of the 5-min window occurring 1 min before the US stimulation). The maximum oxygen consumption rate change (max ΔVO_2_) was calculated using the same method. The maximum ΔRQ was calculated by determining the difference between the lowest RQ observed within the 45–55 min window following the start of US and the baseline metabolic rate, which was defined as the averaged RQ measured within a 28-min window occurring 2 min before the US stimulation. One mouse was excluded from the T_core_ curve in Fig. 2c due to incomplete recording resulting from telemetry sensor failure. The onset was the time point when either T_core_ or VO_2_ was lower than the threshold, defined as the mean value of baseline – 2 × s.d. of the baseline. The end time of the UIH was the time when T_core_ returned back to 34 °C. The total UIH duration was then defined as the time period from the onset to the end of the UIH. An ECG was recorded using the ECGenie system (Mouse Specifics) in awake mice. The heart rate was analyzed using EzCG Signal Analysis Software (v.7.0, Mouse Specifics). To test the UIH effect in different ambient temperatures, we set the temperature in the CLAMS system to be maintained at either ~6 °C, ~22 °C or ~30 °C. Mice were initially adapted to the CLAMS in the preset ambient temperature for 1 d before the experiment. Following the adaptation, US stimulation was delivered to the mice using the same protocol described before when the mice were in the specific ambient temperature (6 °C, 22 °C or 30 °C).

### Temperature measurements in the mouse brain and US transducer probe

The US-induced brain temperature rise was measured by MR thermometry in vivo using a 4.7T MR scanner (Agilent/Varian DirectDrive Console) using the similar method described in the previous paper^34^. Phase images were acquired using a continuously applied gradient-echo imaging sequence with a flip angle of 20°, TR of 10 ms and TE of 4 ms, slice thickness of 1.5 mm and matrix size of 128 × 128 for a 60 × 60 mm field of view. Phase images were processed in real-time using ThermoGuide software (v.1.3.4, Image Guided Therapy) to produce the temperature images based on the proton-resonance frequency shift method. The temperature rise inside the US transducer probe was measured using a fiberoptic thermometer (Luxtron, now LumaSense Technologies). The fiberoptic thermometer was inserted into the US transducer cone and placed on top of the mouse head and beneath the piezoelectric material to measure the temperature rise of the transducer probe.

### Closed-loop feedback control for long-duration UIH

The feedback control system was developed using a feedback controller (MATLAB and an open-source Arduino UNO R3 microcontroller), a sensing element (the telemetry core body temperature sensor) and an actuating device (US). The concept of the closed-loop feedback control system is illustrated in Fig. 3. The feedback control system continuously received T_core_ measurements at 1-min intervals. When the detected T_core_ > T_set_ (T_set_ = 34 °C), the system turned the US on (peak negative acoustic pressure of 1.6 MPa; duty cycle of 50%; pulse repetition frequency of 10 Hz; stimulus duration of 10 s; inter-stimulus interval of 20 s; stimulus number of 6). When T_core_ ≤ T_set_, the US was off. As mice needed to wear the US transducer for a long duration, we designed a special rotary joint cable connector modified from a 360° rotating slip ring (Adafruit) to allow the US transducer cable freely to rotate as the mouse moved around. The total recording time was 30 h and the mice received feedback-controlled US stimulation for 24 h. Water and food were freely accessible to the mice. To compensate for the extra acoustic attenuation resulting from the bubble formation in the US gel after multiple stimulations, the acoustic pressure was increased by 10% and stimulus number was increased to 8 after 12 h of stimulation. The weights of the mice and food in the cage were measured before and after the experiment.

### Fiber photometry recording and data analysis

US-evoked neuronal activities at POA and DMH regions were investigated by fiber photometry recording of GCaMP signals. One month before the recording, AAV5-Syn-GCaMP6s was injected into the POA or DMH region by stereotactic injection using coordinates determined according to the Mouse Brain Atlas: DV = −5.0 mm, AP = 0.2 mm and ML = 0.2 mm for the POA; DV = −5.2 mm, AP = −1.8 mm and ML = 0.2 mm for the DMH. For recording at POA, the US transducer baseplate was attached to the mouse skull with its center hole aligned with the hole drilled during the virus injection. A fiberoptic cannula (MFC_200/245-0.37_6mm_ZF1.25_FLT, Doric Lenses) was implanted approximately 200 µm above the injection site through the hole of the baseplate and the hole was drilled during the virus injection. This design enabled the alignment of the US focal region and the tip of the cannula within the POA. For recording at DMH, a baseplate with a hole at 2 mm off-center was implanted and the cannula was inserted through this hole into the brain. This plate design allowed US stimulation through the center of the plate that was aligned with the POA, while photometry recording was at the DMH region. The cannula and baseplate were then fixed and stabilized by adhesive cement. The mice were then allowed to recover from the surgery for 1 week.

Wearable US transducers for simultaneous US stimulation and fiber photometry recording were built by drilling a 2-mm diameter hole for inserting the optical fiber. The hole was located at the center of the transducer for targeting POA and 2 mm off-center for targeting DMH. The transducer was plugged into the baseplate. A mating sleeve was passed through the hole of the transducer and connected the stainless-steel ferrule to the implanted cannula. The fiber transmitted excitatory blue light (wavelength of 470 nm, power of 4%) and collected GCaMP6s-emitted photons. GCaMP6s signals were collected, digitized and measured with a photometry system (FP3002, Neurophotometrics) in synchronization with US stimulation. An open-source software Bonsai (v.2.7) was used to acquire the photometry data and the synchronization signal from an LED.

Photometry data were acquired before, during and after US stimulation. The acquired data were processed using a method adapted from a previously reported method^56^. Data were first de-bleached using a high-pass filter and converted to a *z* score using the MATLAB ‘zscore’ function. Peaks were identified using the MATLAB ‘findpeaks’ function. The peak amplitude, mean of z score and peak frequency were calculated before US (the 5-s period right before US stimulation), during US (10 s during US stimulation) and after US (the 15-s period right after US stimulation ended). The onset time of the neural activation was determined as the time from the onset of US to the beginning of a successfully evoked Ca^2+^ activity which was defined as *z* score > (mean + 3 × s.d.) of that before US.

Additionally, to verify the positions of the US transducer and the optical fiber, MRI of the mice was conducted using a 9.4T small animal MRI scanner (BioSpec 94/20 USR; Bruker BioSpin MRI, Ettlingen). The mice were anesthetized under 2% isoflurane and placed in an RF volume coil (Bruker BioSpin MRI). T2-weighted fast spin echo images were acquired using the following settings: TR/TE of 2,200/43.53 ms; slice thickness of 0.25 mm; in-plane resolution of 0.125 × 0.125 mm^2^; matrix size of 256 × 256; flip angle of 180°; averages of 8.

### Molecular staining and analysis

Approximately 1.5 h after US stimulation, mice were killed by transcardial perfusion with PBS followed by 4% paraformaldehyde (PFA). Brains were collected and fixed in 4% PFA overnight and equilibrated in 30% sucrose (in 1× PBS) for cryosectioning. The fixed brains were sectioned into 15-µm slices for immunohistochemistry staining and in situ hybridization analysis.

Immunohistochemistry staining was performed using the following primary antibodies: anti-NeuN (Abcam, cat. no. 104225, 1:1,000 dilution), anti-GFAP (Abcam, cat. no. 207165, 1:1,000 dilution), anti-Iba1 (Abcam, cat. no. 178846, 1:1,000 dilution) and anti-c-Fos antibody (Cell Signaling Technology, cat. no. 2250, 1:500 dilution). FISH staining using the RNA-scope assay was performed following the manufacturer’s protocol for RNA-scope Multiplex Fluorescent v2 kit (Advanced Cell Diagnostics (ACD), 323110). The RNA-scope probes used were Mm-FOS (ACD, 316921), Mm-Adcyap1-C2 (ACD, 405911-C2) and Mm-Trpm2-C3 (ACD, 316831-C3).

The stained brain slices were imaged using the multichannel Keyence BZ-X800 microscope with a ×20 objective and BZ-X800 Analyzer software (Keyence Corp). To quantify the percentage of positive cells over different brain regions, the brain slices were registered with the Allen Brain Atlas based on a semi-automatic registration method in MATLAB reported previously^57^. The averaged fluorescence intensity of either protein or RNA markers was calculated within the estimated cell area and determined to be positive if it was higher than mean + 3 × s.d. of the whole-brain background.

### Single-nucleus RNA-sequencing

Approximately 1 h after US stimulation, mice were transcardially perfused with ice-cold phosphate-buffered saline (1× PBS). The POA region from both the US-treated mice (US^+^, *n* = 4) and sham mice (US^−^, *n* = 4) was extracted from each brain and homogenized in a Dounce Homogenizer (Kimble) containing 0.4 ml freshly prepared nuclear extraction buffer (250 mM sucrose, 25 mM KCl, 5 mM MgCl_2_, 10 mM Tris-HCl (pH 8.0), 0.1% Triton X-100 and 0.2 U μl^−1^ recombinant ribonuclease inhibitor (Invitrogen RNaseOUT)) applying four strokes with the A pestle followed by eight strokes with the B pestle. The homogenate of four mice from the control group and four mice from the US group was pooled for nucleus isolation. Samples were filtered through a 30-µm cell strainer tube (Corning Falcon) and centrifuged at 500*g* for 5 min at 4 °C to precipitate nuclei. The dissociated nuclei were washed once and gently resuspended with nuclear storage buffer (PBS with 1% BSA and 0.2 U μl^−1^ recombinant ribonuclease inhibitor). To purify neuron nuclei for single-nucleus RNA-seq, the nuclei suspension was incubated with Alexa Fluor 488 conjugated mouse anti-NeuN antibody (Millipore sigma, MAB377X, 1:200 dilution) for 30 min on ice. After incubation, the nuclei were washed once and resuspended with nuclear storage buffer with 1 μg ml^−1^ DAPI. Samples were filtered through a 30-µm cell strainer tube and shortly sorted using FACS (MoFlo, Beckman Coulter) using FlowJo (v.10.8) software with a specific gating strategy (Supplementary Fig. 1). The neuron nuclei were sorted on DAPI^+^/NeuN^+^ nuclei and collected into 1.5-ml Eppendorf tubes. Approximately 25,000 neuron nuclei were collected from each of the control and US groups and sent to GTAC@MGI for single-cell RNA-seq (10x Genomics). Using a Chromium Next GEM Single Cell 3ʹ GEM, Library and Gel Bead kit v.3.1 (10x Genomics), neuron nuclei were immediately loaded onto a Chromium Single Cell Processor (10x Genomics) for RNA barcoding from single nuclei. Sequencing libraries were constructed according to the manufacturer’s instructions and resulting cDNA samples were run on an Agilent Bioanalyzer using the High Sensitivity DNA Chip to determine cDNA concentrations. The samples were combined and run on an Illumina Nova Seq 6000.

The raw sequencing data were processed using the 10x Genomics CellRanger pipeline (v.6.1.1) based on the reference mouse genome (mm10-2020-A) with default parameters. The data were normalized to the lowest saturated sample, leading to 83,113 reads (mean), 3,207 genes (median) and 7,353 unique molecular identifier (UMI) counts (median) per cell in the control group (*n* = 4 mice). The UIH group (*n* = 4 mice) contained 77,898 reads, 2,998 genes and 6,498 UMI counts per cell.

The Seurat package (v.4.0) in R studio (R v.4.2) was used to process the single-nucleus RNA-seq data. Data were first filtered with the following criteria. The cells that had >5% mitochondrial counts were considered to exhibit extensive mitochondrial contamination and were filtered. Cells that had unique feature counts >7,500 were considered as doublets or multiples and were filtered. Cells that had unique feature counts <200 were also filtered. After filtering, all Seurat objects were merged. The ‘LogNormalize’ method with a scale factor of 10,000 was used for normalization. The ‘FindVariableFeatures’ function was used to extract the top 4,000 variable features. Data were scaled according to mitochondrial percent using the ScaleData function. The IEGs could potentially affect the clustering. Therefore, we removed all 139 IEGs^58^ from the list of feature genes before the following data processing pipeline. Next, principal-component analysis was carried out using the ‘RunPCA’ function. Using the top 40 principal components, the ‘The FindNeighbours’ function and the ‘FindClusters’ were used to identify the initial 34 clusters. Clustering results were visualized using UMAP plots.

Clusters identified as neuronal cells were used for further analysis by checking the average expression level of Kif5c and Camk2a^58^. A total of 21,886 out of 22,612 were identified as neuronal cells. The ‘FindAllMarkers’ function was used to find significant genes in each cluster. The neuronal population was additionally processed to identify neuronal subtypes by repeating the aforementioned methods. Clustering identified 11 excitatory and 24 inhibitory neuronal cell types. Hierarchical tree construction on the excitatory neurons was used to identify the torpor-associated neurons on the basis of the previously reported features, including *Adcyap1*, *Qrfp* and *Esr1* (refs. ^11,12,15^) based on *k*-means clustering. The average expression levels of TRP and PIEZO ion channels, which were summarized from the previous literature^59–61^, were examined across all torpor-associated clusters.

### In vivo knockdown of TRPM2

Lentiviral particles containing *Trpm2* shRNA (m) (cat. no. sc-42675-V, Santa Cruz Biotechnology) were used to knock down the expression of TRPM2 ion channels and lentiviral particles containing scrambled shRNA (m) (cat. no. sc-108080, Santa Cruz Biotechnology) were used as the control. Then, 1.5 µl shRNA solution (10^6^ infectious units of virus) was microinjected to the POA region bilaterally (3 µl total volume). The injection was performed three times every 3 d to ensure sufficient lentiviral particle number in the POA. At 3 weeks after the first injection, the mice received US stimulation at the POA region as previously described. Western blot was performed to evaluate the expression level of TRPM2 in the POA region in the knockdown group and compared to that of the control group injected with scramble shRNA.

### In vitro evaluation of the US sensitivity of TRPM2

To examine the US sensitivity of the TRPM2 ion channel, we used our previously reported in vitro calcium imaging method^34^. HEK293T cells (CRL-3216, ATCC) were grown in DMEM (4.5 g l^−1^ glucose), supplemented with 10% FBS, 2 mM l-glutamine, 100 μg ml^−1^ sodium pyruvate, 1% non-essential amino acids and 1% pen-strep. The TRPM2 ion channel was overexpressed in the cells using Lipofectamine 2000. One day before transfection, cells at 0.5–1 × 10^5^ in 500 μl growth medium without antibiotics cells were seeded in a 48-well plate in every other well. Then, 2 μl of Lipofectamine 2000 (Thermo Fisher Scientific) was first mixed with 0.8 μg *Trpm*2 DNA plasmid (Plasmid 53920, Addgene) in 50 μl Opt-MEM. Then, 50 μl of the complex was added to the cells to allow for 2 d transfection before applying US stimulation. Fluo-4 AM (Thermo Fisher Scientific), a Ca^2+^ indicator, was used to image the spatial dynamics of Ca^2+^ response to US stimulation using a fluorescence microscope at a frame rate of 2.4 frames s^−1^ with the Q-capture (pro 7) software. Functional expression of TRPM2 was validated by observing the Ca^2+^ signal in response to the agonist ADPR (100 μM final concentration, adenosine 5′-diphosphoribose sodium salt, A0752-25MG, Sigma-Aldrich). At 30 s after the start of recording, US stimulation (frequency of 1.7 MHz, acoustic pressure of 1.0 MPa, duty cycle of 40%, PRF of 10 Hz, duration of 60 s) was applied to the cells using the customized US stimulation setup as described in our previous study^34^. We did not use the same US parameter as the in vivo US stimulation protocol due to the difference in the US transducers. The onset time of the activation of TRPM2^+^ cells was defined as the time from the onset of US to the beginning of a successfully evoked Ca^2+^ activity, which was defined as ΔF/F > (mean + 3 × s.d.) of the signal before US stimulation. The temperature of the cell culture chamber was monitored using a fiberoptic thermometer. The tip of the fiberoptic thermometer was inserted at a location approximately 1 mm close to the transducer focus to avoid the interference of US wave propagation. For the blocker study, 5 µl antagonist 2-APB (TOCRIS) was added to 500 μl cell culture solution to a final concentration of 30 μM 1 min before the US stimulation.

### Statistical analysis

Statistics analysis was performed in GraphPad (Prism) and the statistical analysis methods are noted in every figure caption. Statistical differences were considered significant whenever *P* < 0.05.

### Reporting summary

Further information on research design is available in the Nature Portfolio Reporting Summary linked to this article.

## Supplementary information

Supplementary Information Flow cytometry gating strategy.

Reporting Summary

Supplementary Video Representative infrared thermal imaging video of a mouse receiving noninvasive ultrasound stimulation at the POA.

## Data Availability

The data presented in this study are available in the Source data. The original datasets used in the single-nuclei RNA-sequencing analysis can be accessed at NCBI, archived under Gene Expression Omnibus accession code GSE228180. The mice brain atlas used in this study is available in the Allen Brain Atlas. Additional information and requests for resources and reagents that support the findings of this study are available from the corresponding author upon reasonable request. Source data are provided with this paper.

## Source data

Source Data Fig. 1 Statistical Source Data.
Source Data Fig. 2 Statistical Source Data.
Source Data Fig. 3 Statistical Source Data.
Source Data Fig. 4 Statistical Source Data.
Source Data Fig. 6 Statistical Source Data.
Source Data Fig. 7 Statistical Source Data.
Source Data Fig. 8 Statistical Source Data.
Source Data Extended Data Fig. 2 Statistical Source Data.
Source Data Extended Data Fig. 3 Statistical Source Data.
Source Data Extended Data Fig. 4 Statistical Source Data.
Source Data Extended Data Fig. 5 Statistical Source Data.
Source Data Extended Data Fig. 6 Statistical Source Data.
Source Data Extended Data Fig. 7 Statistical Source Data.
Source Data Extended Data Fig. 7 Unprocessed western blots.
Source Data Extended Data Fig. 8 Statistical Source Data.
Source Data Extended Data Fig. 9 Statistical Source Data.
Source Data Extended Data Fig. 10 Statistical Source Data.
