# Supplementary material for: Induction of a torpor-like hypothermic and hypometabolic state in rodents by ultrasound
Source: Nat Metab. 2023 May 25;5(5):789–803. doi: 10.1038/s42255-023-00804-z (PMC10229429; doi:10.1038/s42255-023-00804-z)
Supplement: Supplementary file 1 — Flow cytometry gating strategy. [file 42255_2023_804_MOESM1_ESM.pdf]

nature metabolism

Article

https://doi.org/10.1038/s42255-023-00804-z

Induction of a torpor-like hypothermic and hypometabolic state in rodents by ultrasound

In the format provided by the authors and unedited

Supplementary Fig. 1.

Flow cytometry gating strategy for the control group (US^{-})

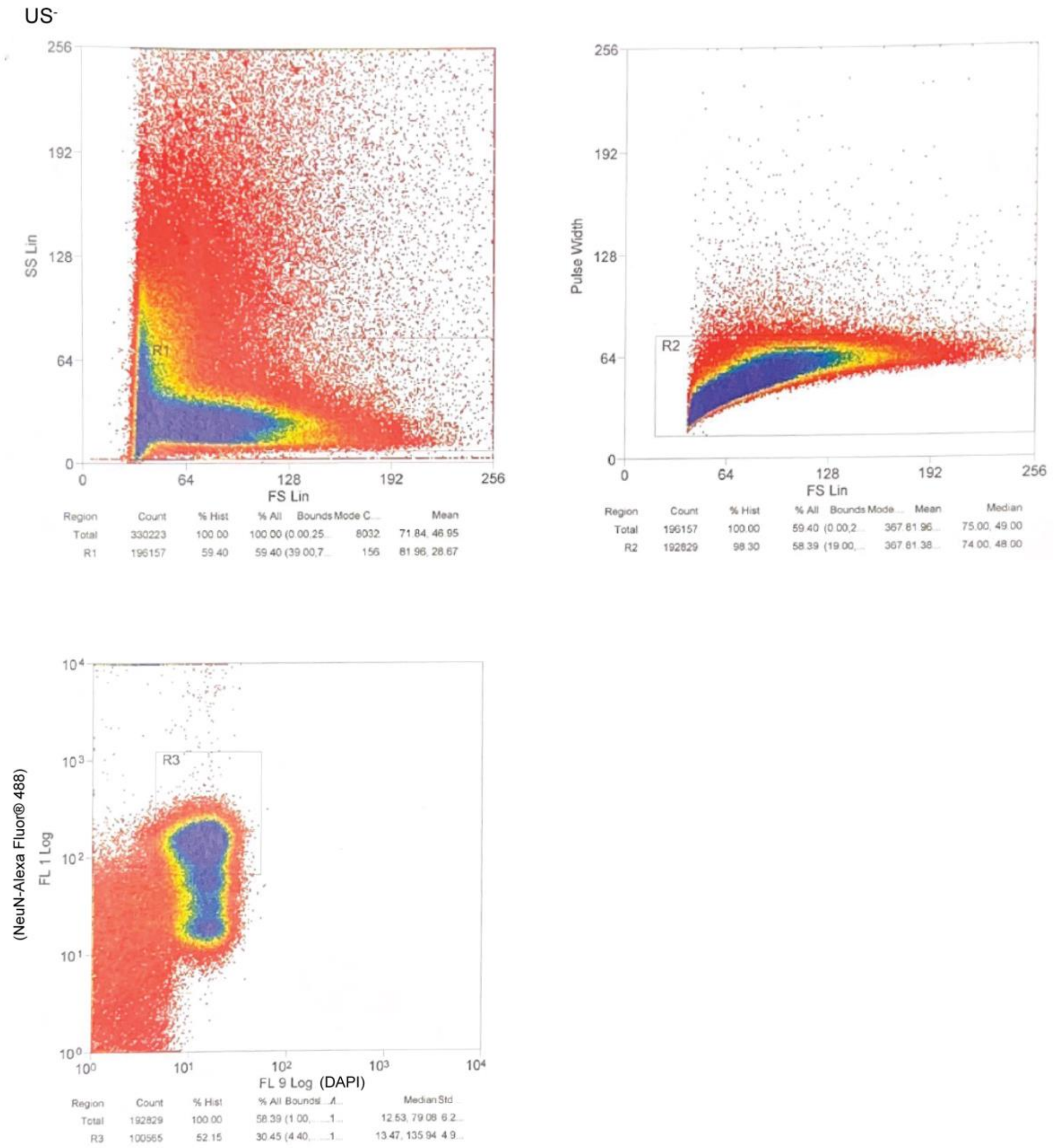

Flow cytometry gating strategy for the ultrasound stimulation group (US^+)

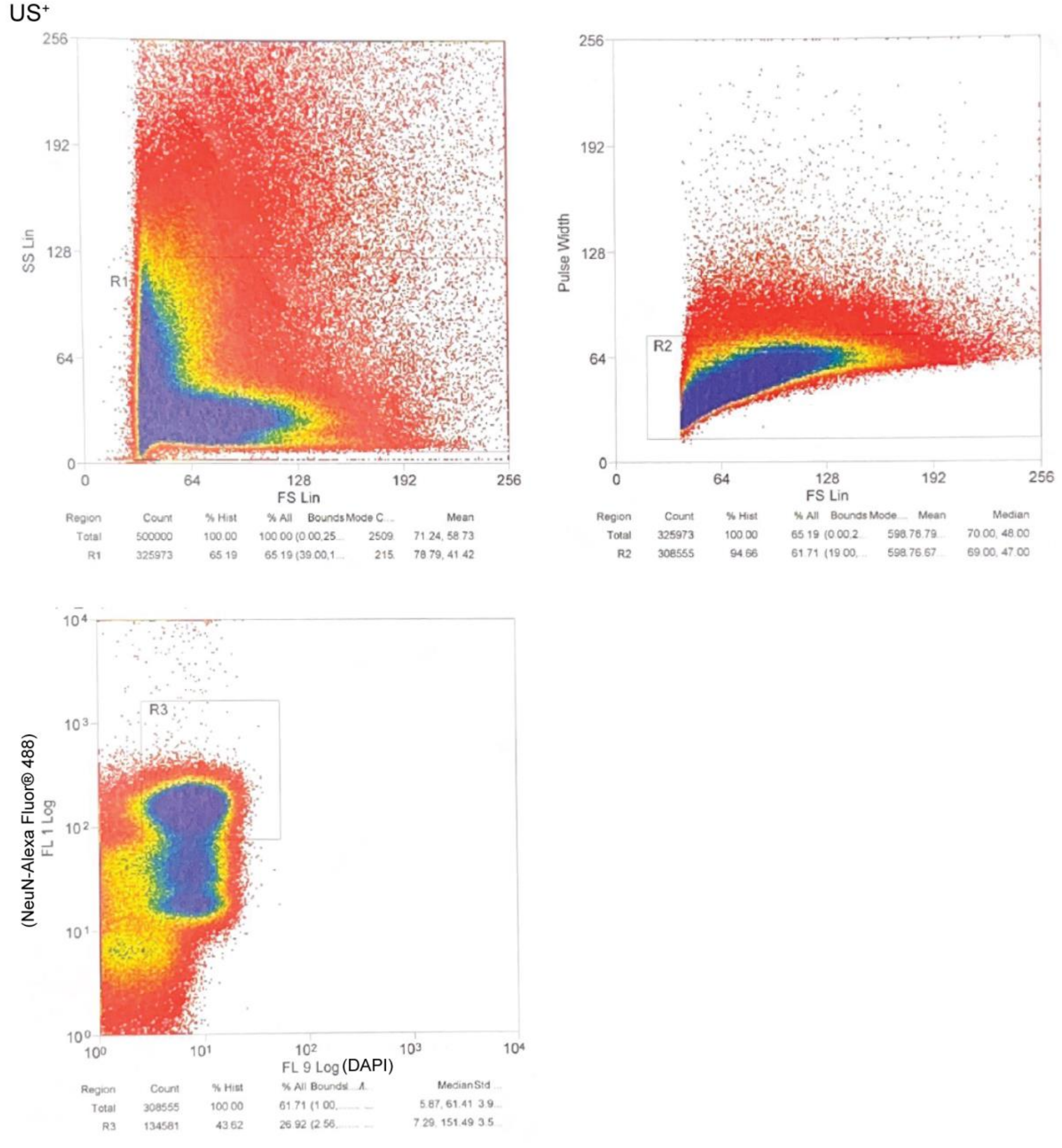

Video S1. Representative infrared thermal imaging video of a mouse receiving noninvasive ultrasound stimulation at the POA.
