# Supplementary material for: Induction of a torpor-like hypothermic and hypometabolic state in rodents by ultrasound
Source: Nat Metab. 2023 May 25;5(5):789–803. doi: 10.1038/s42255-023-00804-z (PMC10229429; doi:10.1038/s42255-023-00804-z)
Supplement: Supplementary file 2 — Reporting Summary [file 42255_2023_804_MOESM2_ESM.pdf]

nature portfolio

Corresponding author(s): Hong Chen

Last updated by author(s): Mar 24, 2023

Reporting Summary

Nature Portfolio wishes to improve the reproducibility of the work that we publish. This form provides structure for consistency and transparency in reporting. For further information on Nature Portfolio policies, see our Editorial Policies and the Editorial Policy Checklist.

Statistics

For all statistical analyses, confirm that the following items are present in the figure legend, table legend, main text, or Methods section.

n/a	Confirmed	
☐	☒	The exact sample size (*n*) for each experimental group/condition, given as a discrete number and unit of measurement
☐	☒	A statement on whether measurements were taken from distinct samples or whether the same sample was measured repeatedly
☐	☒	The statistical test(s) used AND whether they are one- or two-sided<br>*Only common tests should be described solely by name; describe more complex techniques in the Methods section.*
☐	☒	A description of all covariates tested
☐	☒	A description of any assumptions or corrections, such as tests of normality and adjustment for multiple comparisons
☐	☒	A full description of the statistical parameters including central tendency (e.g. means) or other basic estimates (e.g. regression coefficient) AND variation (e.g. standard deviation) or associated estimates of uncertainty (e.g. confidence intervals)
☐	☒	For null hypothesis testing, the test statistic (e.g. F, t, r) with confidence intervals, effect sizes, degrees of freedom and P value noted<br>*Give P values as exact values whenever suitable.*
☒	☐	For Bayesian analysis, information on the choice of priors and Markov chain Monte Carlo settings
☒	☐	For hierarchical and complex designs, identification of the appropriate level for tests and full reporting of outcomes
☒	☐	Estimates of effect sizes (e.g. Cohen's *d*, Pearson's *r*), indicating how they were calculated

Our web collection on statistics for biologists contains articles on many of the points above.

Software and code

Policy information about availability of computer code

Data collection	The commercial software Flir ResearchIR (v4) was used to collect the infrared thermal camera images. The open-source software Bonsai (v2.7) was used to collect the behavior video and the photometry data (with the Neurophotometrics Custom Components Library). The MATLAB (R2021a) was used to collect the telemetry temperature sensor data. CLAX Statistical Software (A commercial software integrated with the CLAMS Comprehensive Lab Animal Monitoring System) was used to collect the metabolic data. The Keyence BZ-X800 software (come with the Keyence microscope) was used to capture the in-situ hybridization and immunofluorescent images. The Q-capture pro 7 was used to record the video of the fluorescent Ca2+ activity of the in-vitro cell culture experiment. ThermoGuide software (v1.3.4) was used to produce the temperature images. FlowJo (v10.8) was used for the flow cytometry experiment.
Data analysis	The commercial software Flir Tool was used to analyze the infrared thermal camera images. Bonsai (2.7) was used to analyze the behavior result and the infrared thermal camera images. MATLAB was used to analyze the behavior result, thermal camera images, core body temperature, metabolic rate VO2, photometry data, all the fluorescent images (in-situ hybridization and immunofluorescent images). R programming language 4.2.0 (R studio, with the Seurat Package) was used to analyze the single-nucleus RNA sequencing data. All the statistic analyze was performed in GraphPad Prism 9.

For manuscripts utilizing custom algorithms or software that are central to the research but not yet described in published literature, software must be made available to editors and reviewers. We strongly encourage code deposition in a community repository (e.g. GitHub). See the Nature Portfolio guidelines for submitting code & software for further information.

Data

Policy information about availability of data

All manuscripts must include a data availability statement. This statement should provide the following information, where applicable:

- Accession codes, unique identifiers, or web links for publicly available datasets
- A description of any restrictions on data availability
- For clinical datasets or third party data, please ensure that the statement adheres to our policy

The data presented in this study is available in the Source data. The original datasets used in the single-nuclei RNA sequencing analysis can be accessed at NCBI, archived under Gene Expression Omnibus accession codes GSE228180. The mice brain atlas used in this study is available in the Allen Brain Atlas. Additional information and requests for resources and reagents that support the findings of this study are available from the corresponding author upon reasonable request.

Human research participants

Policy information about studies involving human research participants and Sex and Gender in Research.

Reporting on sex and gender	N/A
Population characteristics	N/A
Recruitment	N/A
Ethics oversight	N/A

Note that full information on the approval of the study protocol must also be provided in the manuscript.

Field-specific reporting

Please select the one below that is the best fit for your research. If you are not sure, read the appropriate sections before making your selection.

☒ Life sciences ☐ Behavioural & social sciences ☐ Ecological, evolutionary & environmental sciences

For a reference copy of the document with all sections, see nature.com/documents/nr-reporting-summary-flat.pdf

Life sciences study design

All studies must disclose on these points even when the disclosure is negative.

Sample size	Sample size was determined based on similar previously-published studies in the same field. We also confirmed the sample size by performing statistical power analysis based on the estimated standard deviation.
Data exclusions	As stated in the METHOD section, one mouse was excluded from the Tcore curve in Fig. 1C due to incomplete recording resulting from telemetry sensor failure.
Replication	All experiments were reproducible. The mice and rats used for experiments were from multiple litters. The number of mice and rats is presented in the figure caption. The biological replicates or independent experiments times were presented in each figure caption.
Randomization	Animals were randomly allocated into different experimental groups. For the metabolic measurement test, all animals were randomly assigned to the cages in different location.
Blinding	The metabolic measurement study and the brain slices staining were performed single blinded by two different peoples: one for recording/performing experiment and one for analyzing results.

Reporting for specific materials, systems and methods

We require information from authors about some types of materials, experimental systems and methods used in many studies. Here, indicate whether each material, system or method listed is relevant to your study. If you are not sure if a list item applies to your research, read the appropriate section before selecting a response.

Materials & experimental systems

n/a	Involved in the study
☐	☒ Antibodies
☐	☒ Eukaryotic cell lines
☒	☐ Palaeontology and archaeology
☐	☒ Animals and other organisms
☒	☐ Clinical data
☒	☐ Dual use research of concern

Methods

n/a	Involved in the study
☒	☐ ChIP-seq
☐	☒ Flow cytometry
☒	☐ MRI-based neuroimaging

Antibodies

Antibodies used

Immunohistochemistry staining was performed using the following primary antibodies: anti-NeuN (Abcam, Cat: 104225, 1:1000), anti-GFAP (Abcam, Cat: 207165, 1:1000), anti-Iba1 (Abcam, Cat: 178846, 1:1000), and anti-c-Fos antibody (Cell Signaling, Cat: 2250, 1:500). The following RNA scope probes were used: Mm-FOS (ACD #316921), Mm-Adcyap1-C2 (ACD #405911-C2), Mm-Trpm2-C3 (ACD #316831-C3). Anti-NeuN Antibody, clone A60, Alexa Fluor®488 conjugated (MiliporeSigma #MAB377X, 1:200)

Validation

All antibodies were validated by the supplier, please see details below:
anti-NeuN (Abcam, Cat: 104225, 1:1000)
Source / Validation:
It is produced by recombinant technology to ensure better batch-to-batch reproducibility. Knockout edited cell lines were used for gold-standard validation.
Species Reactivity:
Mouse, Rat, Human

anti-GFAP (Abcam, Cat: 207165, 1:1000)
Source / Validation:
Produced recombinantly (animal-free) for high batch-to-batch consistency and long term security of supply. Positive control was performed in Rat hippocampal mixed glia.
Species Reactivity:
Mouse, Rat, Human

anti-Iba1 (Abcam, Cat: 178846, 1:1000)
Source / Validation:
Produced recombinantly (animal-free) for high batch-to-batch consistency and long term security of supply. Positive control was performed in the Rat and mouse normal brain tissues. The negative control data is also provided by the company.
Species Reactivity:
Mouse, Rat, Human

anti-c-Fos antibody (Cell Signaling, Cat: 2250, 1:500)
Source / Purification
Monoclonal antibody is produced by immunizing animals with a synthetic peptide corresponding to residues near the amino terminus of human c-Fos protein.
Specificity / Sensitivity
This antibody detects endogenous levels of total c-Fos protein. The antibody does not cross-react with other Fos proteins, including FosB, FRA1 and FRA2. c-Fos (9F6) Rabbit mAb #2250 non-specifically stains fixed frozen mouse spleen and liver by immunofluorescence.
Species Reactivity:
Human, Mouse, Rat

anti-NeuN antibody (Millipore sigma, MAB377X, 1:200)
Control experiment was conducted to validate the quality of the antibody by the company.
Species Reactivity:
Human, Mouse, Rat

Eukaryotic cell lines

Policy information about cell lines and Sex and Gender in Research

Cell line source(s)

The HEK293T cell line was a gift from X. W. Wang (Washington University in St. Louis) and was originally purchased from ATCC (CRL-3216).

Authentication

None recombinant DNA was engineered into the HEK293T cells. The quality of the cells was checked by the morphology. The endogenous expression of TRPM2 was validated by the TRPM2-agonist ADPR.

Mycoplasma contamination

The HEK293T cells were tested for mycoplasma negative.

Commonly misidentified lines
(See ICLAC register)

The HEK293T cell line is not listed in the database.

Animals and other research organisms

Policy information about studies involving animals; ARRIVE guidelines recommended for reporting animal research, and Sex and Gender in Research

Laboratory animals	Adult (6–9 weeks old) male and female C57BL/6NCrl mice (Charles River Laboratories) were used in this study. UCP1 knockout mice (female, 2–4 months old) were provided by Dr. Jonathan Brestoff's lab. Female and male Wistar Han IGS Rats (Charles River Laboratories, strain code #273) at the age of 4–6 weeks were used in this study. All mice and rats were housed in animal facility at Washington University School of Medicine. Animals were maintained in a temperature-controlled (23 – 26 °C) and humidity-controlled (35 – 65%) environment, with a 12-hour light/dark cycle, and provided with a standard chow diet.
Wild animals	The study did not involve wild animals.
Reporting on sex	Both male and female mice were involved in this study.
Field-collected samples	This study did not involve field-collected samples.
Ethics oversight	All animal studies were reviewed and approved by the Institutional Animal Care and Use Committee (IACUC) of Washington University in St. Louis in accordance with the National Institutes of Health Guidelines for Animal Research (animal protocol number: 21-0187).

Note that full information on the approval of the study protocol must also be provided in the manuscript.

Flow Cytometry

Plots

Confirm that:

☒ The axis labels state the marker and fluorochrome used (e.g. CD4-FITC).

☒ The axis scales are clearly visible. Include numbers along axes only for bottom left plot of group (a 'group' is an analysis of identical markers).

☒ All plots are contour plots with outliers or pseudocolor plots.

☒ A numerical value for number of cells or percentage (with statistics) is provided.

Methodology

Sample preparation	Single cells were isolated from mouse brains with dounce homogenizer
Instrument	MoFlo Flow Cytometer
Software	FlowJo (v10.8)
Cell population abundance	greater than 95% neuron positive cells
Gating strategy	populations were gated with FSC/SSC followed by DAPI+NeuN+

☒ Tick this box to confirm that a figure exemplifying the gating strategy is provided in the Supplementary Information.
