# Supplementary figures and images for: Induction of a torpor-like hypothermic and hypometabolic state in rodents by ultrasound
Source: Nat Metab. 2023 May 25;5(5):789–803. doi: 10.1038/s42255-023-00804-z (PMC10229429; doi:10.1038/s42255-023-00804-z)

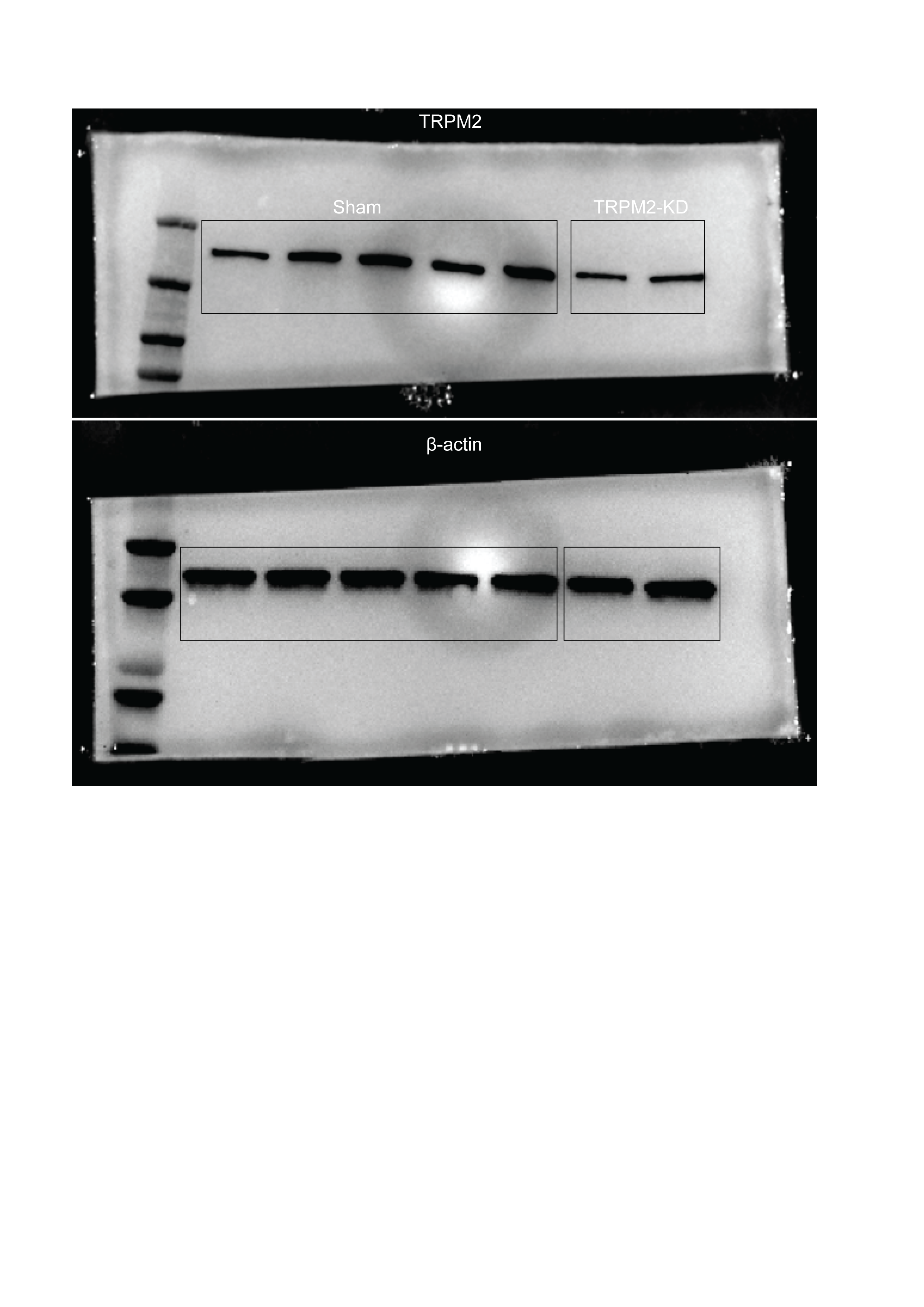

Supplement: Source Data Extended Data Fig. 7 — Unprocessed western blots. [file 42255_2023_804_MOESM17_ESM.tif]
